# Quantitative Analysis of Virus Adsorption and Co-adsorption Behavior Using BET Modeling and SERS Spectroscopy

**DOI:** 10.1021/acs.jpca.5c03375

**Published:** 2025-08-25

**Authors:** Jiaheng Cui, Yanjun Yang, Amit Kumar, Jackelyn Murray, Les Jones, Xianyan Chen, Ralph A. Tripp, Yiping Zhao

**Affiliations:** a School of Electrical and Computer Engineering, College of Engineering, 1355 The University of Georgia, Athens, Georgia 30602, United States; b Department of Physics and Astronomy, The University of Georgia, Athens, Georgia 30602, United States; c Department of Infectious Diseases, College of Veterinary Medicine, The University of Georgia, Athens, Georgia 30605, United States; d Department of Epidemiology & Biostatistics, College of Public Health, The University of Georgia, Athens, Georgia 30602, United States

## Abstract

Understanding virus–surface interactions is essential for developing effective biosensors, diagnostic tools, and antiviral strategies. In this study, we present a systematic investigation of the adsorption and coadsorption behavior of 12 respiratory viruses, including influenza, RSV, coronaviruses, adenovirus, and metapneumoviruses, using surface-enhanced Raman scattering (SERS) on SiO_2_-coated silver nanorod array substrates. Both single viruses (SVs) and binary virus mixtures (2VMs) were analyzed in water and normal human saliva, and spectral data were modeled using a modified Brunauer–Emmett–Teller (BET) adsorption framework. Linear least-squares spectral decomposition enabled the extraction of adsorption coefficients that correlate with virus concentration and surface binding affinity. All viruses exhibited multilayer physisorption consistent with Type II isotherms, with the BET constant *q* varying substantially across virus types. Notably, 2VMs demonstrated a significantly enhanced adsorption behavior, often with *q* values 4–25 times greater than in SVs, indicating strong cooperative or competitive effects. Saliva modulated virus–surface interactions in virus-specific ways, emphasizing the complexity of adsorption dynamics in physiological environments. These findings highlight the limitations of single-virus calibration for quantitative detection in mixed-virus samples and underscore the need for mixture-aware analytical models in biosensing applications. This work provides a robust framework for mechanistic insight and quantitative modeling of virus adsorption relevant to real-world diagnostics and environmental monitoring.

## Graphical abstract

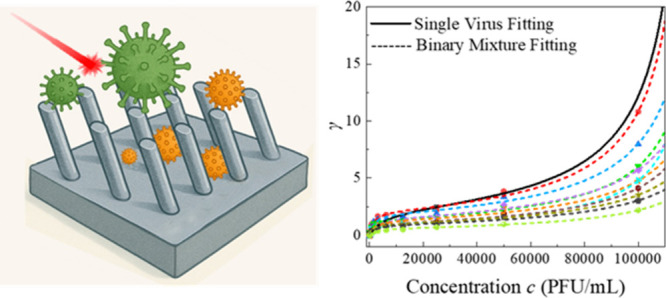

## 1 Introduction

Understanding virus-surface interactions is necessary for various applications, including elucidating the mechanisms of viral infection and enhancing environmental virus removal and detection strategies. Virus adsorption to biological, environmental, or synthetic surfaces is fundamental in processes such as host cell recognition, environmental persistence, and transport through filtration systems. This initial adsorption phase often governs the subsequent fate of the virus, influencing its infectivity, stability, and detectability. Consequently, a comprehensive investigation of virus adsorption behavior is essential for designing effective antiviral coatings, disinfection protocols, environmental monitoring platforms, and novel therapeutic materials.

A growing body of literature has examined virus adsorption across various virus types and substrate materials. These studies have spanned bacteriophages (e.g., MS2, φX-174, Qβ, GA, PP7, UX174, and PR772),^1–4^ enteric viruses like poliovirus, coxsackievirus, rotavirus A, human adenovirus 40,^5–8^ and respiratory viruses such as influenza A (H1N1), influenza B, and adenovirus 2.^9,10^ These studies are instrumental in advancing our understanding of virus behavior in environmental systems, bioprocessing platforms, and biomedical device interfaces. The range of substrates explored includes natural materials such as clay minerals (kaolinite, bentonite), soils, and sediments,^1,2,8^ engineered surfaces like chromatographic media and functionalized nanofibers,^7,11,12^ and biologically relevant interfaces including cell monolayers, biofilms, and wastewater solids.^3,6,13^ These systems are highly diverse, mirroring the broad applicability of virus adsorption research across environmental virology, biosensing, water treatment, and pharmaceutical science.

Experimental conditions across studies have spanned a wide range of pH values, typically from acidic (pH 5) to alkaline (pH 9.5), with lower pH generally enhancing virus adsorption by minimizing electrostatic repulsion.^2,8,14^ Ionic strength, commonly modulated through buffer systems and salt concentrations up to 1.5 M NaCl, often modulates electrostatic interactions and virus-surface affinity.^11,15,16^ Temperature effects, ranging from 4 to 37 °C, influence adsorption kinetics and virus stability.^17,18^ Virus-surface interactions are typically assessed using batch or column adsorption assays,^6,18,19^ Surface plasmon resonance (SPR) and quartz crystal microbalance (QCM-D) for real-time binding analysis,^4,9,10^ and advanced microscopy techniques such as total internal reflection fluorescence (TIRF) microscopy and atomic force microscope (AFM) for nanoscale resolution of virus attachment.^20,21^

Various models have been employed to describe virus adsorption behavior. The Langmuir isotherm is frequently used for systems showing monolayer, saturable adsorption.^3,4,9^ In contrast, the Freundlich isotherm is preferred for heterogeneous surfaces.^7,12,15^ Linear isotherms are observed where adsorption scales proportionally with concentration, such as virus retention in sludge.^6^ More sophisticated models, including multistep kinetic and random sequential adsorption frameworks, have been proposed to capture irreversible or layered adsorption behaviors in systems such as papillomavirus on epithelial cells or adenovirus on membranes.^10,13,22^

A variety of factors influence virus adsorption, including surface charge, hydrophobicity, pH, ionic strength, and the presence of dissolved organic matter or competing molecules. Adsorption is often enhanced at lower pH and higher ionic strength, conditions that favor electrostatic attraction.^4,15^ Substrate hydrophobicity and steric effects can enhance or impede virus binding depending on the virus capsid structure.^12,20^ Additionally, dissolved organic matter and humic substances frequently reduce virus adsorption by competing for binding sites or masking surface functionalities.^6,8^

While many studies have examined the adsorption behavior of individual viruses, relatively few have investigated the coadsorption of multiple viruses simultaneously. For example, Riordan et al. measured the adsorption of bacteriophages PP7, UX174, and PR772 on the same surface, revealing differences in binding affinity but not explicitly addressing competition.^11^ Armanious et al. explored adsorption of bacteriophages MS2, fr, GA, and Qβ on identical surfaces, hinting at preferential adsorption patterns.^4^ Similarly, Hébrant et al. compared GA and Qβ binding to drinking water biofilms, showing distinct kinetics without direct coadsorption experiments.^3^ Pisharody et al. examined bacteriophages MS2, SUSP2, and Rotavirus A in parallel on different substrates but did not test multivirus competition,^7^ and Yin et al. suggested potential competition in wastewater matrices without directly analyzing mixed-virus scenarios.^6^

To address this critical knowledge gap, the present study systematically investigates the adsorption and coadsorption behavior of 12 clinically relevant respiratory viruses, spanning influenza A/B, respiratory syncytial viruses (RSV A/B), coronaviruses, adenovirus, and human metapneumoviruses—using surface-enhanced Raman scattering (SERS) on SiO_2_-coated silver nanorod (AgNR@SiO_2_) substrates. By incorporating both single-virus (SV) samples and binary virus mixtures (2VMs), this work provides the first quantitative and comparative assessment of virus–surface interactions under both controlled aqueous and physiologically relevant (saliva) conditions. Importantly, we combine label-free spectroscopic measurements with a modified Brunauer–Emmett–Teller (BET) adsorption model to extract meaningful physical parameters, such as the BET constant, that describe the strength and cooperativity of virus adsorption. The study also employs spectral decomposition to resolve individual virus contributions in complex mixtures, enabling the investigation of competitive and synergistic effects that modulate adsorption in coinfection scenarios. Through this integrated approach, we aim to elucidate the physical and biochemical determinants of virus adsorption on nanostructured surfaces, and to advance the development of robust, mixture-aware calibration frameworks for SERS-based virus detection platforms.

## 2 Experiments and Data Analysis

### 2.1 Materials

Silver (99.999%, Kurt J. Lesker, Jefferson Hills, PA, USA) and titanium pellets (99.995%, Kurt J. Lesker, Jefferson Hills, PA, USA) were purchased as evaporation materials. Tetraethylorthosilicate (TEOS; 99.9%, Alfa Aesar, Ward Hill, MA, USA), ammonium hydroxide (28.0–30.0 wt %, J. T. Baker, Phillipsburg, NJ, USA) and ethanol (EtOH; 95%, Sigma-Aldrich, St. Louis, MO, USA) were used for silica coating on AgNRs. Polydimethylsiloxane (PDMS; Sylgard 184 silicone elastomer kit) was purchased from Dow Corning (Midland, MI, USA). Dulbecco’s Modified Eagle Medium (DMEM; GIBCO BRL Laboratories, Grand Island, NY, USA) supplemented with 1% fetal bovine serum (FBS; Hyclone Laboratories, Salt Lake City, UT, USA) was used for virus culture. Deionized water (DI water) with a resistivity of 18.2 MΩ·cm was used throughout all the experiments. All the reagents were used without further purification.

### 2.2 Design of Virus Mixture Specimens

The following 12 types of single viruses (SVs) were used in the study: adenovirus type 5 (Ad5), SARS-CoV-2 (WA1/2020, CoV-2), SARS-CoV-2 B1.1.7 variant (CoV-2 B1), human coronavirus 229E (CoV-229E), human coronavirus OC43 (CoV-OC43); influenza A H1N1 Brisbane (H1N1), influenza A H3N2 Hong Kong (H3N2), and influenza B (Flu B); human metapneumovirus from strain A (HMPV-A) and B (HMPV-B); as well as respiratory syncytial virus from strain A2 (RSV-A2) and B1 (RSV-B1). These viruses were selected due to their significant impact on respiratory health and their known cocirculation potential, leading to coinfections that can exacerbate disease severity. Literature surveys confirm that influenza, RSV, and coronaviruses frequently cocirculate, posing serious public health risks, particularly in the context of severe respiratory illnesses.^23–27^ For SV measurements, each virus was prepared at 12 different concentrations ranging from 50 to 10^5^ PFU/mL.

To study virus coadsorption and assess model generalization, four clinically relevant 2VMs, i.e., influenza virus and RSV viruses, were designed (see Table S1). Virus combinations were selected based on biological interactions, spectral distinctiveness, and public health relevance.^23–27^ According to literature, the diameter of the influenza virus is about 80–120 nm,^28^ while the size of the RSV virus is about 150–300 nm.^29^ Each 2VM included 12 concentration levels for Virus A and 12 for Virus B, resulting in a complete factorial design of 144 unique mixtures covering all (*c*_A_, *c*_B_) combinations from 50 to 10^5^ PFU/mL.^30^

To evaluate the influence of the sample matrix, all virus specimens (SVs and 2VMs) were prepared in deionized (DI) water and human saliva. DI water served as a controlled medium for baseline adsorption behavior, while saliva was chosen for its clinical utility as a noninvasive, self-collectible, and stable diagnostic matrix.^31–33^ Saliva samples were collected from healthy, asymptomatic adult donors with no recent respiratory illness and were confirmed virus-free via RT-PCR assays.

### 2.3 Virus Incubation

All viruses were propagated in Vero E6 cells, which were maintained in DMEM supplemented with 1% FBS. Briefly, cells were infected using a multiplicity of infection (MOI) = 0.1. After 48 h, the viruses were harvested in serum-free DMEM, followed by freeze–thawing, after which the contents were collected and centrifuged at 4,000 g for 15 min at 4 °C. The virus titers were similar, i.e., 10^6^ PFU/mL, as determined by plaque assay as previously described.^34–36^ The reference specimen for these studies was diluted in DMEM supplemented with 1% FBS. Influenza strains, H1N1 and H3N2, were propagated in embryonated chicken eggs, and virus titers were determined by hemagglutination assay using chicken red blood cells. The influenza virus titers ranged between 10^7^ - 10^8^ 50% egg infectious dose (EID_50_). The reference specimen for these studies was naïve allantoic fluid. All the experiments on SARS-CoV-2 and SARS-CoV-2 variants were conducted in a biosafety level 3 (BSL-3) lab, while others were performed in a BSL-2 lab. All the experimental operations should follow the biosafety guidelines:https://www.cdc.gov/coronavirus/2019-nCoV/lab/lab-biosafety-guidelines.html.

### 2.4 AgNR@SiO_2_ Array SERS Substrate Fabrication

AgNR@SiO_2_ array SERS substrates were prepared by the oblique angle deposition (OAD) and salinization via hydrolysis of TEOS as described previously.^37,38^ OAD is selected for its capability to produce uniform, reproducible nanorod structures that significantly enhance SERS sensitivity. The AgNR substrates were first prepared using OAD.^39,40^ Glass slides (0.5 in. × 0.5 in.), cleaned with piranha solution (3:1 sulfuric acid to hydrogen peroxide), were mounted in a custom-designed electron beam deposition system. A 20 nm titanium adhesion layer and a 100 nm silver film were sequentially deposited at 0.2 and 0.3 nm/s, respectively. Then, the vapor incident angle was adjusted to 86°, and a thickness of 2000 nm Ag film was deposited at a rate of 0.3 nm/s to form the AgNRs on the substrates. The entire evaporation process was conducted under high vacuum conditions (chamber pressure <3 × 10^–6^ Torr). Following deposition, the AgNR substrates were immersed in a homogeneous solution containing 30 mL of ethanol, 4 mL of DI water, and 500 μL of TEOS under stirring for 20 min. The coating of SiO_2_ was initiated after adding 560 μL of ammonium hydroxide. The substrates were removed from the reaction solution after 5 min, followed by DI water rinsing and N_2_ drying. This process was designed to achieve a uniform, conformal ∼ 2 nm SiO_2_ coating, thin enough to maintain substantial plasmonic enhancement while improving substrate stability. According to our previous electron microscope study, the average spacing between the adjacent SiO_2_-coated AgNRs was ∼ 140 nm.^38,41^ To verify the reproducibility, uniformity, and enhancement performance of the SERS substrate used in this study, we have included a standardized quality control procedure detailed inSectionS2. The resulting substrates exhibited a high SERS enhancement factor, suitable for sensitive virus detection. Subsequently, arrayed small wells (4 wells, each with a diameter of 4 mm and a depth of 1 mm) on a PDMS layer were molded on the AgNR@SiO_2_ array to restrict the effective sensing areas,^42^ and we refer to them as AgNR@SiO_2_ wells.

### 2.5 SERS Measurement

Twenty μL of each virus specimen was dispensed onto the AgNR@SiO_2_ wells and incubated for 15 min. Given the dense media and saliva, each well was washed with DI water and air-dried at 20 °C. Then, the SERS spectra were acquired using a Tec5USA Raman spectrometer (Tec5USA Inc.) equipped with a 785 nm excitation laser with a beam diameter of ∼ 100 μm, a power of 35 mW, and an acquisition time of 1 s. The rationale of using 785 nm as our wavelength can be found inSectionS2. Approximately 350 SERS spectra were collected from different substrate locations for each type of virus specimen for the spectral averaging process.

### 2.6 SERS Spectrum Pretreatment

SERS spectral preprocessing was performed in several steps. First, cosmic rays, or highly energetic particle features, were removed from the raw spectra, and each spectrum was then cropped to the spectral range of 320–2500 cm^–1^. A Gaussian–Lorentzian fitting-based baseline correction was applied,^43^ with spectral featureless regions (320–400 cm^–1^ and 1875–2500 cm^–1^) used to guide baseline estimation. After baseline removal, spectra were further cropped to the region of interest (450–1700 cm^–1^) to exclude noninformative regions. Each spectrum was then normalized by its average intensity to standardize across samples. Finally, average spectra were calculated for each virus concentration in the single-virus data sets and for each concentration pair in the binary virus mixtures.

### 2.7 Spectral Least-squares Fittings

Spectral least-squares fittings were performed to quantitatively analyze the normalized spectra. For SV decomposition in water, the normalized spectrum *I*_*c*_A__(Δν) of virus A at concentration *c*_*A*_ was modeled using linear decomposition in ref 44 as

$$I_{c_{A}}\left(\Delta \nu \right)=a\left(c_{A}\right)I_{A}\left(\Delta \nu \right)+\left(1-a\left(c_{A}\right)\right)I_{bg}\left(\Delta \nu \right)+\epsilon \left(\Delta \nu \right)$$ 1

where *I*_*A*_(Δν) represents the average normalized spectrum of virus A at the highest concentration (PFU/mL PFU/mL, approximating the true virus spectrum), *I*_*bg*_(Δν) represents the average normalized spectrum of the DI water on AgNR@SiO_2_ background, and *a*(*c*_*A*_) is the linear coefficient constrained between 0 and 1, expected to increase monotonically with virus concentration *c*_*A*_. The term ϵ­(Δν) was modeled as independent Gaussian noise.

For 2VM involving viruses A and B at the concentration combination of (*c*_*A*_, *c*_*B*_) in water, the decomposition was expanded as

$$\begin{array}{c} I_{c_{A},c_{B}}\left(\Delta \nu \right)=a\left(c_{A},c_{B}\right)I_{A}\left(\Delta \nu \right)+b\left(c_{A},c_{B}\right)I_{B}\left(\Delta \nu \right)\\ +\left(1-a\left(c_{A},c_{B}\right)-b\left(c_{A},c_{B}\right)\right)I_{bg}\left(\Delta \nu \right)+\epsilon \left(\Delta \nu \right) \end{array}$$ 2

where coefficients *a*(*c*_*A*_,*c*_*B*_) and *b*(*c*_*A*_,*c*_*B*_) are the linear coefficients constrained between 0 and 1, and that *a*(*c*_*A*_,*c*_*B*_) + *b*(*c*_*A*_,*c*_*B*_) ≤ 1 to ensure the sum of three coefficients is 1.

For SVs and 2VMs in saliva, the normalized spectrum of saliva *I*_*saliva*_(Δν) should be added as another decomposition component, soeqs [Disp-formula eq1] and 2 should be modified to

$$\begin{array}{c} I_{c_{A}}\left(\Delta \nu \right)=a\left(c_{A}\right)I_{A}\left(\Delta \nu \right)+b\left(c_{A}\right)I_{saliva}\left(\Delta \nu \right)+\left(1-a\left(c_{A}\right)-b\left(c_{A}\right)\right)I_{bg}\left(\Delta \nu \right)+\epsilon \left(\Delta \nu \right) \end{array}$$ 3

and

$$\begin{array}{c} I_{c_{A},c_{B}}\left(\Delta \nu \right)=a\left(c_{A},c_{B}\right)I_{A}\left(\Delta \nu \right)+b\left(c_{A},c_{B}\right)I_{B}\left(\Delta \nu \right)+c\left(c_{A},c_{B}\right)I_{saliva}\left(\Delta \nu \right)\\ +\left(1-a\left(c_{A},c_{B}\right)-b\left(c_{A},c_{B}\right)-c\left(c_{A},c_{B}\right)\right)I_{bg}\left(\Delta \nu \right)+\epsilon \left(\Delta \nu \right) \end{array}$$ 4

where all linear coefficients should be constrained between 0 and 1.

Coefficients for each concentration or concentration combination were determined using least-squares optimization.^45^ All computational analyses were conducted using Python 3.11.5, leveraging key libraries including NumPy 1.24.3,^46^ Pandas 2.2.1,^47^ Matplotlib 3.8.4,^48^ SciPy 1.11.1,^49^ and Scikit-learn 1.4.1.^50^ Specifically, the least-squares optimization was executed using the SciPy library’s “minimize” function with the Sequential Least Squares Programming algorithm.^51^ All calculations were performed on a workstation equipped with an Intel Core i7–13700KF CPU (3.40 GHz) and 64 GB RAM.

## 3 Results and Discussion

Figure 1 shows the experimental workflow for our SERS-based virus adsorption study. The workflow involved preparing SV and 2VM specimens in DI water and saliva at varying concentrations, followed by SERS measurements on AgNR@SiO_2_ substrates. Each specimen, whether SV or 2VM, was dispensed onto the AgNR@SiO_2_ substrate and allowed to adsorb under controlled conditions. After washing and drying, the substrate was subjected to Raman spectroscopy to collect SERS spectra. The average SERS spectra were computed from multiple replicates. A linear combination model was applied to decompose mixed-virus spectra into contributions from individual virus reference spectra and the substrate background. The linear coefficients corresponding to each virus spectrum were extracted using least-squares fitting and interpreted as proxies for relative surface adsorption. Adsorption behavior was then modeled using a modified BET relationship for SVs and each component in 2VMs to evaluate competitive or cooperative adsorption effects. Goodness-of-fit metrics (e.g.,*R* ^2^) were used to assess reconstruction accuracy.

**Figure 1:**
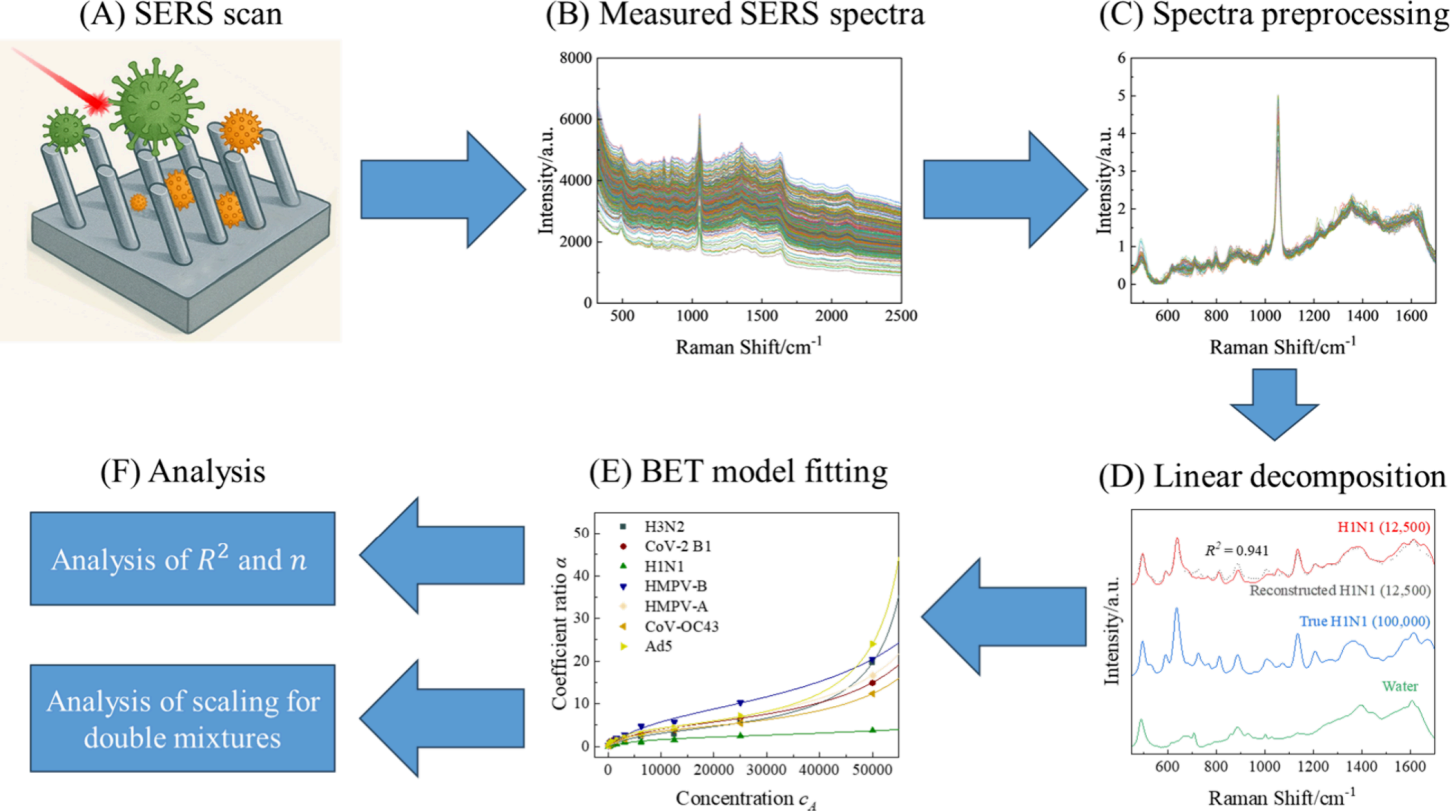
Schematic illustration of the experimental and analysis procedure: Step A: Collection of SERS spectra from individual viruses and virus mixture specimens; Step B: Removal of cosmic ray artifacts and initial cropping of the spectra to the 320–2500 cm^–1^ range; Step C: Spectral preprocessing including Gaussian–Lorentzian function baseline removal, further cropping to 450–1700 cm^–1^, and average intensity normalization; Step D: Linear decomposition of the average spectra for each virus at various concentrations; Step E: Fitting the linear decomposition coefficient ratios versus concentration using a modified Brunauer–Emmett–Teller (BET) model; and Step F: Analysis of the fitting parameters and discussion/explanation of results.

### 3.1 SERS Spectra of Virus and Mixtures

Figure 2A and the lower four spectra in Figure 2B display representative average SERS spectra of 12 individual respiratory viruses at high viral concentration (e.g., 10^5^ PFU/mL): Ad5, CoV-2, CoV-229E, CoV-2 B1, CoV-OC43, Flu B, HMPV-A, HMPV-B, H1N1, H3N2, RSV-A2, and RSV-B1. Major peak assignments for these virus spectra are provided in refs 30 and 52. While these SERS spectra share several common vibrational features, they are distinguishable by unique characteristic peaks specific to each virus. As highlighted in the shaded regions of Figure 2A, nearly all viruses exhibit two or three characteristic peaks between 600–770 cm^–1^, primarily associated with guanine vibrational modes. In addition, broad spectral bands are observed between 1200–1650 cm^–1^, corresponding to various vibrational modes of proteins and nucleic acids. Beyond these standard features, individual viruses also show distinct peaks. For example, Figure 2B shows that RSV-A2 exhibits a unique peak at Δν = 1052 cm^–1^ (blue dash-dotted line), while RSV-B1 displays an additional peak at Δν = 793 cm^–1^ (green dash-dotted line). Both H1N1 and H3N2 present multiple prominent peaks, including those at Δν = 495, 639, 886, 1135, and 1203 cm^–1^, as indicated by the red dashed lines in Figure 2B. Viruses of the same type or family tend to have highly similar spectral profiles. For instance, RSV-A2 and RSV-B1, H1N1, H3N2, and Flu B; HMPV-A and HMPV-B; as well as CoV-2, CoV-229E, CoV-2 B1, and CoV-OC43, each form visually consistent spectral groups. In contrast, viruses from different families display significant spectral differences, enabling their distinction based on SERS spectra. Significantly, these viral spectral signatures differ from the background signal of the DI water, which is also shown in Figure 2A for comparison.

**Figure 2:**
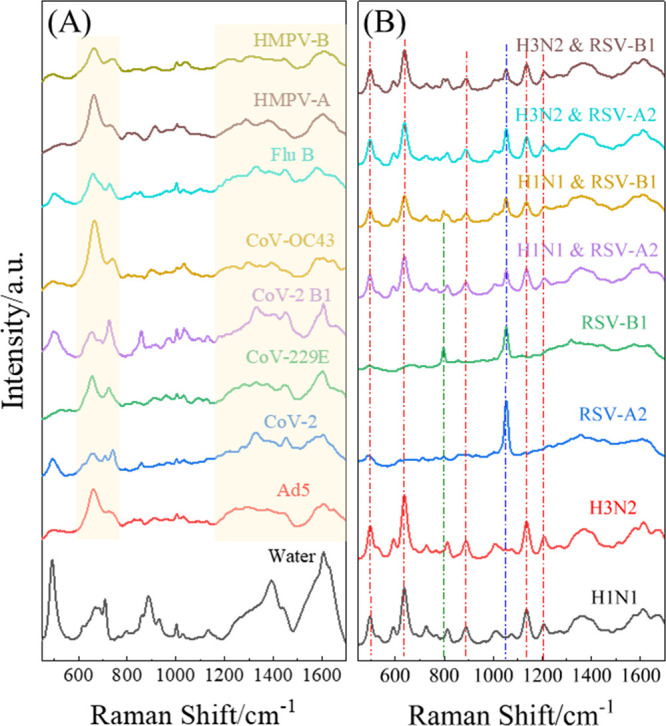
(A) Average spectra of eight individual viruses measured at 10^5^ PFU/mL in water, together with the water spectrum. (B) Average spectra of H1N1, H3N2, RSV-A2, and RSV-B1 at 10^5^ PFU/mL and their corresponding 2VMs, with each viral concentration at 10^5^ PFU/mL.

Figure 2B shows representative average SERS spectra of four Flu and RSV 2VMs: H1N1 & RSV-A2, H3N2 & RSV-A2, H1N1 & RSV-B1, and H3N2 & RSV-B1, each at 10^5^ PFU/mL. These spectra appear quite similar overall, primarily due to the close spectral resemblance between RSV-A2 and RSV-B1 and between H1N1 and H3N2. However, because RSV and Flu viruses possess distinct spectral features, each 2VM spectrum exhibits characteristic peaks from both virus types. As highlighted by the dash-dotted lines in Figure 1B, all four 2VM spectra contain distinguishable contributions from influenza and RSV viruses, as indicated by the dash-dotted lines with different colors. The main differences among the four spectra lie in the relative intensities of these peaks, reflecting subtle variations in each virus pair’s composition and coadsorption behavior.

### 3.2 Spectral Decomposition by the Least-Squares Method

Figure 3A shows a representative least-squares fitting for H1N1 at 12,500 PFU/mL diluted in DI water, as well as the H1N1 spectrum (at 10^5^ PFU/mL, blue) and water spectrum (green) used. The high similarity between the measured spectrum (red) and reconstructed spectrum (gray dashed) demonstrates the effectiveness of the linear combination approach, yielding a coefficient of determination *R* ^2^ = 0.941. Key spectral features from the reference H1N1 spectrum are clearly preserved in both the measured and reconstructed spectra, particularly the characteristic peaks at Δν = 593, 637, 1135, and 1363 cm^–1^. The measured and reconstructed spectra also have characteristic peaks at Δν = 488 and 1606 cm^–1^, which match the background spectrum. Inspection of the residuals reveals that three virus-derived peaks at Δν = 723, 889, and 1003 cm^–1^ are slightly overpredicted, whereas the broad band from 1476 to 1686 cm^–1^ for water is under-predicted. This systematic misfit suggests that (1) subtle intensity shifts in the virus Raman bands at high concentration and (2) potential subtle nonlinear interactions between the virus and substrate at this concentration could not be fully captured by the linear combination model. Complete comparisons between experimental and fitted spectra across all concentrations for each virus are available in the GitHub link provided in Section S2. Visual inspection of these fits reveals consistently strong agreement between measured and reconstructed spectra across the concentration range tested. At higher concentrations (≥50,000 PFU/mL), virus-specific peaks are prominently captured in the reconstructions, with only minor deviations in peak intensities. At lower concentrations (≤781 PFU/mL), while the signal-to-noise ratio decreases, the reconstructions still effectively capture the underlying spectral features, though with slightly reduced accuracy in some cases where viral signatures approach detection limits. Across all 12 SV data sets, Figure 3B presents boxplots of their *R* ^2^ distributions (whiskers = ± 1.5 IQR, boxes = 25th-75th percentiles, center line = median, dots = mean). The median *R* ^2^ for each virus lies between 0.933 and 0.981, with an overall median of 0.970. Even the lowest-performing virus (H3N2 at 198 and 391 PFU/mL) maintains *R* ^2^ > 0.735, and only two spectra dip below 0.800. These uniformly high *R* ^2^ values confirm that our reference library yields robust fits for single-virus spectra.

**Figure 3:**
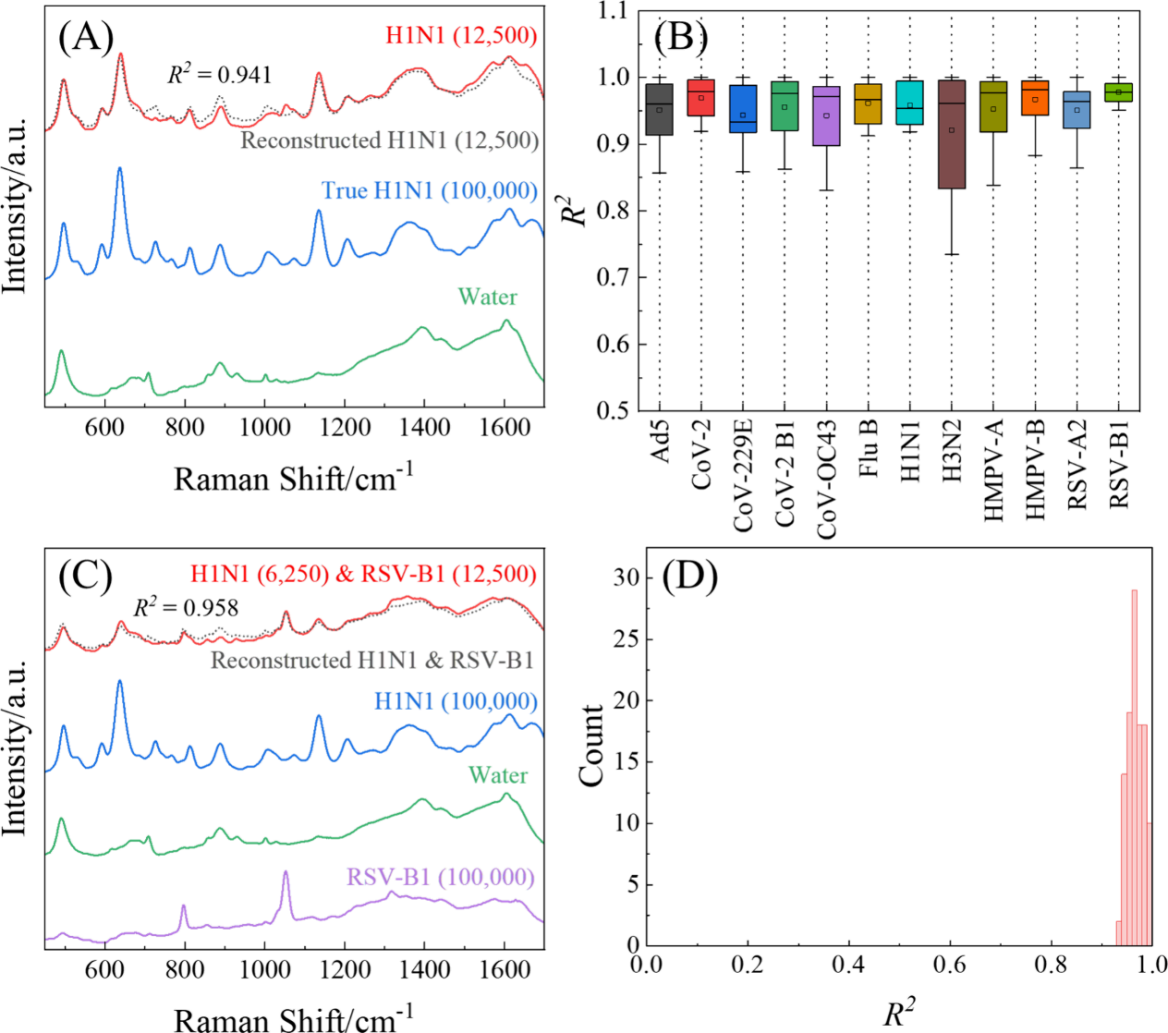
Linear decomposition fitting and goodness-of-fit analysis for SV and 2VM SERS spectra. (A) Example of linear fitting for H1N1 at 12,500 PFU/mL: true spectrum (red), reconstructed spectrum (gray dashed), and reference spectra for H1N1 (10^5^ PFU/mL, blue) and water (green). (B) Boxplot of *R* ^2^ values from fittings of all SV spectra; boxes indicate interquartile range (IQR), whiskers show ± 1.5 × IQR, center lines mark medians, and dots represent means. (C) Linear fitting for a 2VM of H1N1 (6,250 PFU/mL) and RSV-B1 (12,500 PFU/mL): true spectrum (red), reconstructed spectrum (gray dashed), and reference spectra for H1N1 (blue), RSV-B1 (purple), and water (green). (D) Histogram of *R* ^2^ values for the H1N1 & RSV-B1 mixture fittings.

The decomposition was then extended to 2VMs using eq[Disp-formula eq2]. Figure 3C depicts the fit for H1N1 (6,250 PFU/mL) & RSV-B1 (12,500 PFU/mL). Here, three references (H1N1 at 10^5^ PFU/mL, blue; RSV-B1 at 10^5^ PFU/mL, purple; water, green) combine to reconstruct the measured spectrum (red) with *R* ^2^ = 0.958. The close agreement between the measured and reconstructed spectra confirms that the 2VM spectrum can be effectively modeled as a linear combination of the individual virus and substrate components. Key spectral features are well preserved in the reconstruction, including H1N1 characteristic peaks at Δν = 637 and 1136 cm^–1^, RSV-B1 signatures at Δν = 801 and 1053 cm^–1^, and the water peak at Δν = 488 cm^–1^. Residual analysis again highlights a slight overprediction of RSV-B1 bands between 825 and 1019 cm^–1^ and under-prediction of RSV-B1 (1308–1405 cm^–1^) and H1N1 (1447–1589 cm^–1^) features, consistent with minor concentration-dependent spectral shifts. The complete set of 2VM reconstructions (available in Section S2) shows that the linear decomposition approach maintains high accuracy across different virus combinations and concentration ratios. The reconstructions successfully capture the dominant spectral features of both viruses, though with slightly reduced accuracy for the lower-concentration component. This consistent performance across diverse mixture compositions demonstrates the robustness of our linear decomposition method for analyzing complex viral samples. Figure 3D shows a histogram of *R* ^2^ values for all H1N1 & RSV-B1 concentration pairs. The distribution is tightly clustered around 1.0 (mean = 0.968, SD = 0.015). Comparable histograms for the other three binary virus combinations appear in Figure S1 and likewise exhibit high *R* ^2^ values (0.963 ± 0.018 for H1N1 & RSV-A2, 0.958 ± 0.021 for H3N2 & RSV-A2, and 0.969 ± 0.017 for H3N2 & RSV-B1). These consistently high *R* ^2^ values across all mixtures demonstrate that even in the presence of overlapping spectral features, linear least-squares decomposition reliably captures the contributions of each component across all mixture ratios.

Similar to virus in water, we verified that our linear least-squares decomposition remains robust in the complex matrix of saliva. As shown in Figure S2A, a representative reconstructed spectrum for the H1N1 saliva sample (12,500 PFU/mL, gray) closely matches the measured spectrum (red). The reconstruction successfully captures key spectral features from all three components: H1N1 viral peaks at Δν = 590, 637, 810, 887, 1135, and 1206 cm^–1^; saliva matrix signatures at Δν = 1032, 1259, and 1446 cm^–1^; and water contributions at Δν = 488, 710, 1393, and 1606 cm^–1^. Closer examination of the residuals reveals minor discrepancies, including a slight underestimation of the H1N1 peak at Δν = 1470 cm^–1^ and the saliva peak at Δν = 1034 cm^–1^. Some regions show overestimation, particularly around Δν = 685 cm^–1^ (contributed primarily by H1N1) and between 1340 and 1424 cm^–1^ (where H1N1 exhibits more pronounced features than the flatter profiles of saliva and water in this region). Despite the slight overestimation or underestimation, the linear combination approach effectively incorporates all components into the reconstruction. Figure S2B presents boxplots of *R* ^2^ values for all ten SV in saliva spectra: seven viruses achieve median *R* ^2^ > 0.95, while three exhibit relatively lower fitting quality: CoV229E (median *R* ^2^ = 0.901; minimum *R* ^2^ = 0.804 at 195 PFU/mL), H1N1 (median *R* ^2^ = 0.917; two low *R* ^2^s of 0.583 at 391 PFU/mL and 0.679 at 781 PFU/mL), and H3N2 (median *R* ^2^ = 0.932; minimum *R* ^2^ = 0.781 at 391 PFU/mL). Overall, the *R* ^2^ distributions for most viruses in saliva are comparable to those observed in water, with only CoV-229E and H1N1 showing modestly reduced fitting quality. This slight reduction is likely linked to the increased spectral complexity in the saliva matrix, particularly in regions where viral and saliva signatures overlap. Nevertheless, the consistently high *R* ^2^ values across different viruses and concentrations demonstrate that our linear decomposition approach maintains its effectiveness even in complex biological matrices, with sufficient robustness to reliably detect and quantify viral components amid the background of saliva constituents.

As shown in Figure S2C, the representative reconstructed spectrum for a 2VM in saliva closely matches the measured spectrum with *R* ^2^ = 0.956. The reconstruction successfully captures numerous spectral features from all four components with varying degrees of accuracy. Well-recovered peaks include H1N1 signatures at Δν = 590, 637, 810, and 887 cm^–1^; the characteristic saliva peak at Δν = 1003 cm^–1^; and the water peak at Δν = 488 cm^–1^. Several features show underestimation, including H1N1 contributions from 1219 to 1478 cm^–1^; RSV-B1 peaks at Δν = 798 and 1319 cm^–1^; and saliva/water features at Δν = 1034 and 1032 cm^–1^. Conversely, overestimated features include the H1N1 peak at Δν = 1135 cm^–1^ and the RSV-B1 peak at Δν = 1051 cm^–1^. These variations suggest that binary mixtures in saliva have much more complex spectral interactions between matrix and virus components than in water or single viruses.

We also examined the *R* ^2^ histograms for each 2VM in Figure S2D–G. Unlike the near-Gaussian distributions in water (Figure 3D), 2VMs in saliva with H1N1 display pronounced asymmetries: H1N1 & RSV-A2 is slightly left-skewed, i.e., clustering at high *R* ^^2^^ with a long tail toward lower values (mean = 0.922, SD = 0.061, minimum = 0.750); H1N1 & RSV-B1 remains left-skewed (mean = 0.943, SD = 0.028, minimum = 0.872). 2VMs in saliva with H3N2 are more symmetrical. H3N2 & RSV-A2 exhibits a bell-shaped distribution (mean = 0.934, SD = 0.032, minimum = 0.865); and H3N2 & RSV-B1 shows an approximately bell-shaped curve except for a plateau around 0.92–0.94 (mean = 0.922, SD = 0.061, minimum = 0.750). These distribution patterns confirm saliva’s biochemical complexity introduces greater variability in fit quality, particularly for H1N1-containing mixtures. Despite these variations and the minor systematic residuals observed in some spectra, the linear least-squares method provides robust reconstructions for both SV and 2SM SERS spectra. The consistently high *R* ^2^ values across diverse sample types establish a solid quantitative framework for downstream analysis of adsorption behavior in coinfection systems. All fitting comparison plots of SVs and 2VMs are available in the GitHub link provided in Section S2 **.**

### 3.3 Modeling SERS Intensity with Virus and Background Adsorption on Hotspots

Assume that there are *N*_hs_ total number of hot spots in a laser beam. The laser beam diameter is *A*_*l*_. For SV in water, the number of adsorbed viruses on the hot-spots within *A*_*l*_ is *N*_*A*_, the number of background molecules on the hot-spots within *A*_*l*_ is *N*_bk_, where the background could be contamination in water or molecular compositions in saliva. Originally, all hot-spots are covered by background molecules. Therefore, the SERS intensities for the virus and background can be expressed as,

$$\begin{array}{c} \{\begin{array}{c} I_{A}=G_{A}N_{A}\sigma_{A}FI_{0}\\ I_{\mathrm{bk}}=G_{\mathrm{bk}}N_{\mathrm{bk}}\sigma_{\mathrm{bk}}FI_{0} \end{array} \end{array}$$ 5

where *G*_*A*_ and *G*_bk_ are the enhancement factors, σ_*A*_ and σ_bk_ are the Raman scattering cross sections for virus A and background molecule, respectively; while *F* represents the instrument and collection efficiency, and *I*_0_ is the intensity of the excitation laser. Thus, the total SERS intensity of the SV can be expressed as

$$\begin{array}{c} I_{SERS}=G_{A}N_{A}\sigma_{A}FI_{0}+G_{\mathrm{bk}}N_{\mathrm{bk}}\sigma_{\mathrm{bk}}FI_{0} \end{array}$$ 6

If we assume that *N*_bk_ is a constant, i.e., the virus is adsorbed on an already-contaminated SERS substrate, and let us assume that both σ_*v*_ and σ_bk_ are normalized, i.e.,

$$\begin{array}{c} \int \sigma_{A}d\left(\Delta \nu \right)=\int \sigma_{\mathrm{bk}}d\left(\Delta \nu \right)=1 \end{array}$$ 7

Then the normalized SERS spectrum can be written as

$$\begin{array}{c} I_{\mathrm{SERS}}^{N}=\frac{I_{\mathrm{SERS}}}{\int I_{\mathrm{SERS}}d\left(\Delta \nu \right)}=\frac{G_{A}N_{A}\sigma_{A}+G_{\mathrm{bk}}N_{\mathrm{bk}}\sigma_{\mathrm{bk}}}{G_{A}N_{A}+G_{\mathrm{bk}}N_{\mathrm{bk}}} \end{array}$$ 8

Comparing eq 8 to eq 1, we have

$$\begin{array}{c} a=\frac{G_{A}N_{A}}{G_{A}N_{A}+G_{\mathrm{bk}}N_{\mathrm{bk}}},1-a=\frac{G_{\mathrm{bk}}N_{\mathrm{bk}}}{G_{A}N_{A}+G_{\mathrm{bk}}N_{\mathrm{bk}}} \end{array}$$ 9

In general, *G*_*A*_ ≠*G*_bk_. Let

$$\begin{array}{c} N_{A}=K_{A}f_{A}\left(c_{A}\right) \end{array}$$ 10

where *K*_*A*_ is an adsorption coefficient, while *f*_*A*_(*c*_*A*_) represents the virus adsorption behavior on the surface. Let $\alpha =\frac{a}{1-a}$ , we can obtain

$$\begin{array}{c} \alpha \propto \frac{G_{A}}{G_{\mathrm{bk}}}f_{A}\left(c_{A}\right) \end{array}$$ 11

i.e., the α–*c*_*A*_ relationship represents the adsorption behavior of virus A.

For 2VM of viruses A and B in water, the SERS spectrum can be written as

$$\begin{array}{c} I_{\mathrm{SERS}}=G_{A}N_{A}\sigma_{A}FI_{0}+G_{B}N_{B}\sigma_{B}FI_{0}+G_{\mathrm{bk}}N_{\mathrm{bk}}\sigma_{\mathrm{bk}}FI_{0} \end{array}$$ 12

The normalized SERS spectrum can be written as,

$$\begin{array}{c} I_{\mathrm{SERS}}^{N}=\frac{G_{A}N_{A}\sigma_{A}+G_{B}N_{B}\sigma_{B}+G_{\mathrm{bk}}N_{\mathrm{bk}}\sigma_{\mathrm{bk}}}{G_{A}N_{A}+G_{B}N_{B}+G_{\mathrm{bk}}N_{\mathrm{bk}}} \end{array}$$ 13

Therefore,

$$\begin{array}{c} \{\begin{array}{c} a=\frac{G_{A}N_{A}}{G_{A}N_{A}+G_{B}N_{B}+G_{\mathrm{bk}}N_{\mathrm{bk}}}\\ b=\frac{G_{B}N_{B}}{G_{A}N_{A}+G_{B}N_{B}+G_{\mathrm{bk}}N_{\mathrm{bk}}}\\ 1-a-b=\frac{G_{\mathrm{bk}}N_{\mathrm{bk}}}{G_{A}N_{A}+G_{B}N_{B}+G_{\mathrm{bk}}N_{\mathrm{bk}}} \end{array} \end{array}$$ 14

Let *N*_*A*_ = *K*’_*A*_ *f*_*A*_^’^(*c*_*A*_,*c*_*B*_), *N*_*B*_ = *K*’_*B*_ *f*_*B*_^’^(*c*_*A*_,*c*_*B*_), $\alpha^{’}=\frac{a}{1-a-b},\beta^{’}=\frac{b}{1-a-b},\gamma =\frac{a}{b}$ . Based on eq 14, we have,

$$\begin{array}{c} \{\begin{array}{c} \alpha^{’}\propto f_{A}^{’}\left(c_{A},c_{B}\right)\\ \beta^{’}\propto f_{B}^{’}\left(c_{A},c_{B}\right)\\ \gamma \propto \frac{f_{A}^{’}\left(c_{A},c_{B}\right)}{f_{B}^{’}\left(c_{A},c_{B}\right)} \end{array} \end{array}$$ 15

For coadsorption, if there is no competing adsorption effect, then *f*_*A*_^’^(*c*_*A*_,*c*_*B*_) ∝ *f*_*A*_(*c*_*A*_) regardless of the concentration of virus B, and vice versa. If there are competing adsorptions, the *f*_*A*_^’^(*c*_*A*_,*c*_*B*_) – *c*_*A*_ relationship will depend on *c*_*B*_; similarly, the *f*_*B*_^’^(*c*_*A*_,*c*_*B*_) – *c*_*B*_ relationship will depend on *c*_*A*_. These derivations can be extended to SVs and 2VMs in saliva.

### 3.4 Single Virus Adsorption

Figure 4 plots the obtained α as a function of viral concentration*c*_*A*_ for 12 different respiratory viruses suspended in aqueous solution. The overall shapes of most α–*c*_*A*_ curves are S-shaped, characteristic of cooperative or multilayer adsorption behavior. Figure 4A shows adsorption profiles for seven viruses: Ad5, CoV-2 B.1, CoV-OC43, H1N1, H3N2, HMPV-A, and HMPV-B, each exhibiting two distinct turning points. At low concentrations, the curves are convex, indicating rapid surface adsorption. As the concentration increases, the curves become concave, reflecting a saturation regime consistent with multilayer formation. In contrast, Figure 4B displays data for five other viruses, Flu B, RSV-A2, RSV-B1, CoV-2, and CoV-229E, showing convex-to-concave transitions. However, these transitions are abrupt, and some data exhibit greater scatter. Nevertheless, all 12 viruses demonstrate adsorption behaviors consistent with Type II isotherms.

**Figure 4:**
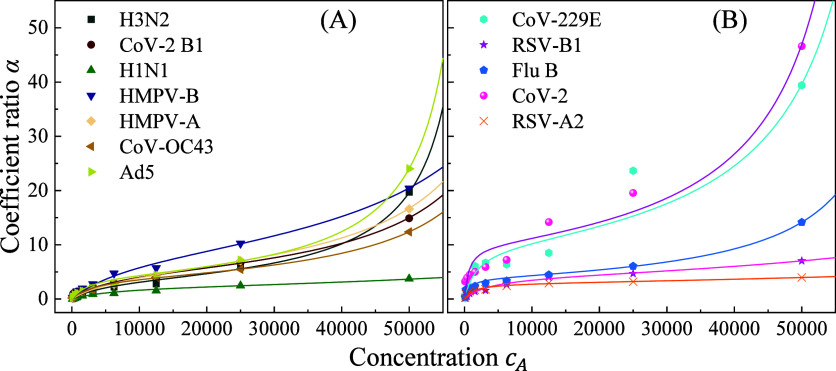
Plots of α as a function of viral concentration *c*_*A*_ for 12 respiratory viruses in water: (A) Ad5, CoV2B1, CoV-OC43, H1N1, H3N2, HMPV-A, and HMPV-B, with relative small *q* values; and (B) Flu B, RSVA2, RSVB1, CoV2, and CoV229E with large *q* values.

Type II isotherms are commonly associated with multilayer physical adsorption on heterogeneous or macroporous surfaces.^53^ In such systems, a monolayer forms first via weak van der Waals interactions, followed by the buildup of additional layers. This contrasts with the monolayer-limited Langmuir model. To quantitatively describe these adsorption behaviors, the data were fit using a modified BET equation,^53^

$$\begin{array}{c} \alpha =\frac{Aqkc_{A}}{\left(1-kc_{A}\right)\left[1+\left(q-1\right)kc_{A}\right]} \end{array}$$ 16

where *q* is the BET constant related to the heat of adsorption of the first layer vs the heat of liquefaction, $k=\frac{1}{c_{A}^{0}}$ is the concentration scaling factor, *c*_*A*_^0^ is the saturation concentration, and *A* is a proportionality constant reflecting the maximum adsorption capacity.

The solid curves in Figure 4 represent the fitted curves using this model, with all fits exhibiting excellent agreement (the coefficients of determination). The good fit supports the dominance of multilayer physisorption oversimple monolayer adsorption. Fitting parameters are summarized in Table S2. Among them, the BET constant*q* emerges as a key parameter characterizing virus-surface interaction strength. Viruses in Figure 4A (Ad5 to HMPV-B) show lower *q* values (11.8 to 24.1), suggesting relatively weak first-layer binding. H3N2 shows the lowest interaction (*q* = 11.8), whereas Ad5 binds more strongly (*q* = 24.1). In contrast, viruses in Figure 4B display significantly higher*q* values (36.2 to 120.4). CoV-229E and RSV-B1 are on the lower end of this range, while RSV-A2 and the original SARS-CoV-2 strain exhibit the strongest interactions (*q* = 120.4 and 104.5, respectively). While *q* values vary substantially across viruses, two other key parameters show more consistency: the proportionality constant *A* (indicating maximum adsorption capacity) and the saturation concentration *c*_*A*_^0^. Across all viruses, *A* values range from 2.85 to 10, while *c*_*A*_^0^ varies between 6 × 10^4^ and 1.7 × 10^5^ PFU/mL. This suggests that while the maximum adsorption capacity and saturation point remain relatively consistent, the adsorption strength captured by *q* is highly virus-dependent.

A closer examination of the virus-specific*q* values provides insights into how structural and biochemical features influence adsorption behavior in water. H3N2 has the weakest adsorption among influenza viruses, followed by H1N1, with *q* values of 11.8 and 19.9, respectively. In contrast, Flu B exhibits a much stronger interaction with a *q* of 98.6. This trend suggests that virus type (Influenza A vs B) plays a more significant role than subtype (H1 vs H3) in determining adsorption strength. The enhanced adsorption of Flu B may be attributed to differences in envelope composition or glycoprotein architecture that promote stronger binding to the substrate. A similarly broad spectrum of adsorption strength is observed among coronaviruses. The original SARS-CoV-2 strain demonstrates the highest surface affinity with a *q* of 104, whereas CoV-229E and CoV-OC43 exhibit moderate *q* values of 36.2 and 21.98, respectively. Notably, the B.1 variant of SARS-CoV-2 shows a substantially lower *q* of 14.5, indicating weaker nonspecific binding to the surface. Among RSV strains, A2 (*q* = 120.4) binds more strongly than B1 (*q* = 39.8), potentially reflecting G protein or glycan composition differences.

These findings highlight the virus-dependent nature of adsorption behavior, which is likely governed by envelope structure, surface protein properties, and glycosylation patterns. Differences between virus types (e.g., Flu A vs Flu B, RSV-A2 vs RSV-B1, or SARS-CoV-2 vs CoV-229E) have a greater impact on *q* than differences between subtypes or variants. Such insights are essential for optimizing surface-based detection technologies like SERS, where adsorption efficiency directly affects signal quality.

Figure 5 extends this analysis to virus adsorption in saliva. Figure 5A includes Flu B, Ad5, RSV-B1, HMPV-B, HMPV-A, CoV-229E, and RSV-A2, which show low *q* values (4 to 23.2). Figure 5B includes H3N2, CoV-OC43, and H1N1, with much higher *q* values (62.3 to 160). As in aqueous media, the BET model fits the data well (*R* ^2^ > 0.925), except for H3N2 and H1N1 (*R* ^2^ = 0.762 and 0.800), where adsorption may be more variable. Compared to water-based results, saliva alters virus-surface interaction energetics. For the viruses in Figure 5A, *q* values drop markedly (e.g., Flu B: 98.6 → 4; RSV-A2:120.4 → 23.2), indicating reduced adsorption strength. In contrast, *q* values for Figure 5B viruses increase significantly (e.g., H1N1:19.9 → 160). These shifts likely reflect biochemical effects of saliva, including surface conditioning by proteins and mucins, changes in viral aggregation or hydration, and competitive binding. For viruses like Flu B and RSV-A2, suppressing *q* in saliva suggests interference by saliva components, such as mucins or enzymes, which may shield viral adhesion sites or occupy binding sites on the substrate. Conversely, increased *q* for H3N2, H1N1, and CoV-OC43 may indicate that saliva promotes conformational changes or enhances electrostatic compatibility, improving surface affinity. The low *R* ^2^ observed for H3N2 and H1N1 may stem from increased heterogeneity in saliva or breakdown of the BET model assumptions under physiological conditions.

**Figure 5:**
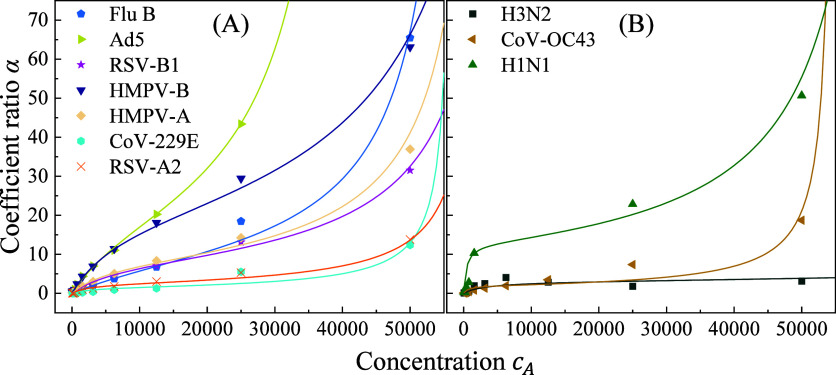
Plots of α as a function of viral concentration *c*_*A*_ for 10 respiratory viruses in saliva: (A) Flu B, Ad5, RSV-B1, HMPV-B, HMPV-A, CoV-229E, and RSV-A2, with relatively small *q* values; and (B) H3N2, CoV-OC43, and H1N1 with large *q* values.

The single virus adsorption behavior is highly sensitive to viral structure and environmental context. The contrast between water and saliva highlights the need to consider physiological complexity when designing and interpreting surface-based detection methods.

### 3.5 Binary-Virus Coadsorption

We investigated the adsorption characteristics of four representative binary virus mixtures in water to explore potential coadsorption effects. Despite a second virus, all experimental adsorption curves for individual viruses in these mixtures exhibited clear BET-type behavior, consistent with multilayer physisorption. For example, Figure 6A,B presents the adsorption coefficient γ_*H*1*N*1_ as a function of H1N1 concentration *c*_*H*1*N*1_ at fixed concentrations of RSV-A2*c*_*RSV*–*A*2_, and γ_*RSV*–*A*2_ as a function of RSV-A2*c*_*RSV*–*A*2_ at fixed concentrations of *c*_*H*1*N*1_, respectively, for the mixture H1N1 & RSV-A2 in water. The corresponding adsorption curves α–*c*_*A*_ for single-virus systems in water are also included for reference. Regardless of the concentrations of the second virus (*c*_*B*_) in the mixture, all γ_*A*_–*c*_*A*_ relationships conform to BET-type adsorption. The dashed curves in Figure 6A,B show the fitting results using the BET equation, with most exhibiting high coefficients of determination (*R* ^2^ > 0.9). Only four out of 19 curves have slightly lower *R* ^2^ values between 0.86 and 0.89 (see Figure S3A,B in SI), indicating good model agreement overall. Similar BET behavior was observed for the other virus mixtures (see Figure S4 in SI), confirming that the dominant mechanism remains physisorption even in the presence of multiple virus types.

**Figure 6:**
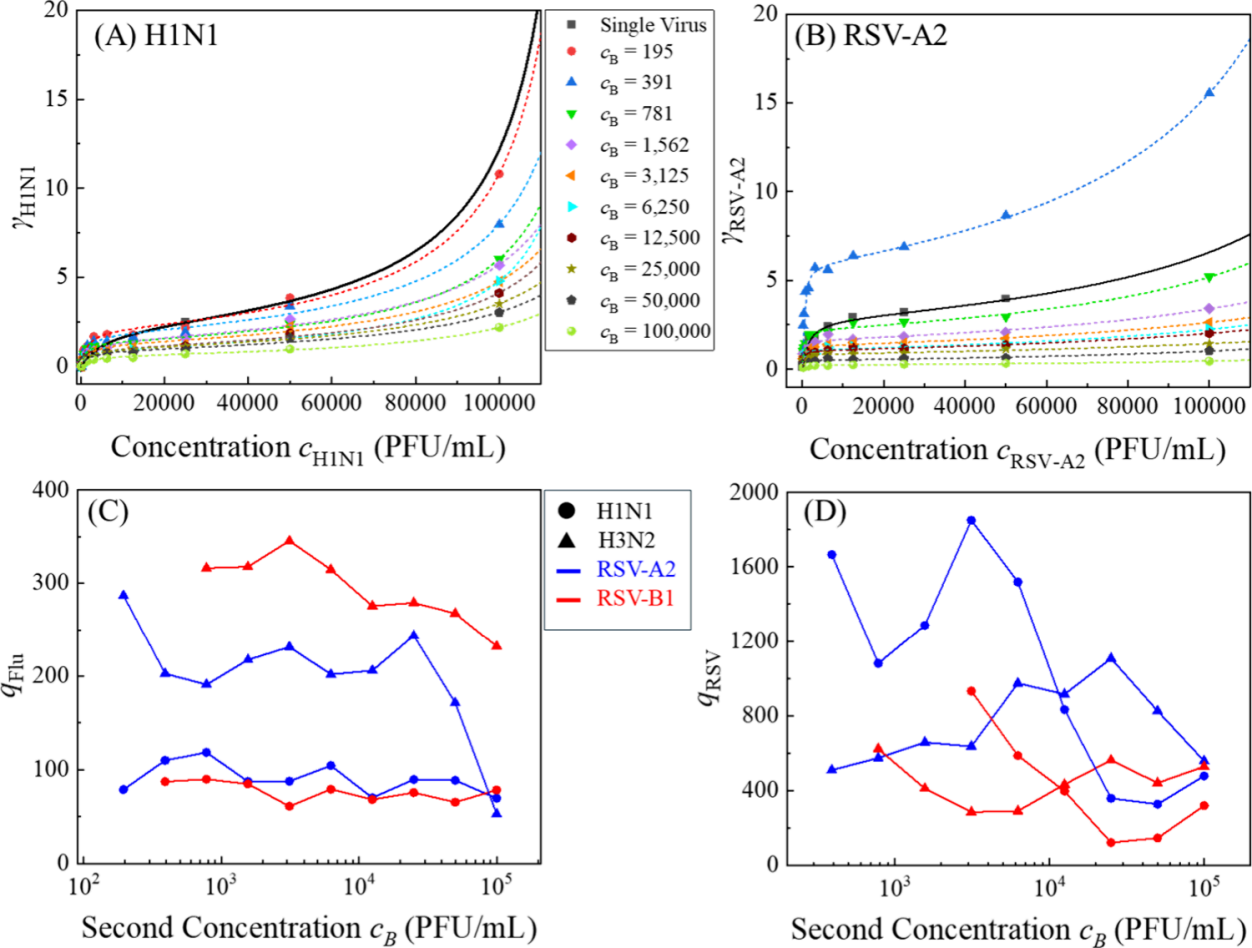
Plots of (A) γ_*H*1*N*1_ versus the concentration *c*_*H*1*N*1_ for fixed concentrations *c*_*RSV*–*A*2_ and (B) γ_*RSV*–*A*2_ versus the concentration *c*_*RSV*–*A*2_ for fixed concentrations of *c*_*H*1*N*1_ for the mixture H1N1 & RSV-A2 in water. All the dashed curves are the fitting based on the BET equation, and the black solid curves are the α–*c*_*A*_ relationship for the corresponding single virus in water. The plots of extracted *q* values based on the BET equation for (C) H1N1 (●) and H3N2 (▲) as a function of RSV concentration and (D) RSV-A2 (blue) and RSV-B1 (red) as a function of Flu concentration.

While BET behavior is preserved, the BET constant *q*, changes significantly in the presence of a second virus. Figure 6C summarizes the*q* values of H1N1 and H3N2 in mixtures with RSV. For H1N1, the BET constant remains relatively stable in both mixtures, with an average ${\mathrm{q̅}}_{H1N1}=90.5$ and standard deviation σ_*H*1*N*1_ = 16 in the RSV-A2 mixture, and ${\mathrm{q̅}}_{H1N1}=76.7$ , σ_*H*1*N*1_ = 10 in the RSV-B1 mixture. In contrast, H3N2 exhibits much greater variation: in the RSV-A2 mixture, ${\mathrm{q̅}}_{H3N2}=200$ with σ_*H*3*N*2_ = 61, and in the RSV-B1 mixture, to ${\mathrm{q̅}}_{H3N2}=294$ with σ_*H*3*N*2_ = 36. These values are markedly higher than those observed in the single-virus cases, where *q*_*H*1*N*1_ = 19.9 and *q*_*H*3*N*2_ = 11.9, representing enhancements of 4–4.5 times for H1N1 and 17–25 for H3N2.

A similar trend is observed for RSV viruses in these mixtures, as shown in Figure 6D. For RSV-A2, the average ${\mathrm{q̅}}_{RSV-A2}=750$ with σ_*RSV*–*A*2_ = 211 in the H1N1 mixture and ${\mathrm{q̅}}_{RSV-A2}=1044$ with σ_*RSV*–*A*2_ = 578 in the H3N2 mixture. RSV-B1 shows a slightly more stable behavior, with ${\mathrm{q̅}}_{RSV-B1}=447$ , σ_*RSV*–*B*1_ = 121 in the H1N1 mixture; and ${\mathrm{q̅}}_{RSV-B1}=417$ , σ_*RSV*–*B*1_ = 305 in the H3N2 mixture. Compared to their single-virus counterparts, *q*_*RSV*–*A*2_ = 120 and *q*_*RSV*–*B*1_ = 40, these results represent 6–9-fold increases for RSV-A2 and up to 10-fold increases for RSV-B1.

Another key parameter in the BET equation is*k*, which predicts the saturation concentration *c* ^0^. As shown inFigures S3E,F, the *k* values for each virus remain relatively consistent across different mixture compositions, with most variations staying within the same order of magnitude. The only notable exception is H3N2 (10^5^ PFU/mL) in the H3N2 & RSV-A2 mixture, which substantially increases from 1.51 × 10^–7^ to 5.36 × 10^–7^. Importantly, we observe that for any given virus, th e*k* values remain similar regardless of which virus is the second component in the mixture. All calculated *k* values correspond to *c* ^0^ values exceeding 10^5^ PFU/mL (see Figure 3G,H), indicating that even our highest experimental concentration has not reached saturation. This observation aligns with the absence of asymptotic behavior in the BET curves around 10^5^ PFU/mL, confirming that additional binding sites remain available even at the highest concentrations tested.

We further extended this investigation to 2VM adsorption in saliva. Figure 7A,B presents the adsorption coefficient γ_*H*1*N*1_ and γ_*RSV*–*A*2_ as functions of their respective concentrations in the H1N1 & RSV-A2 mixture in saliva. The curves again display BET-type behavior, with most fits achieving *R* ^2^ > 0.9 (see Figure S5A,B in SI). Consistent BET behavior was also observed in the other 2VMs in saliva (see Figure S6 in SI).

**Figure 7:**
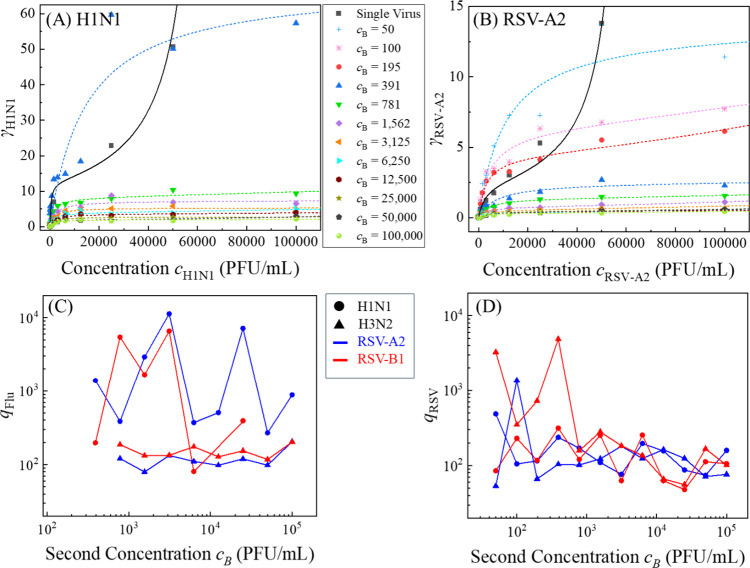
Plots of (A) γ_*H*1*N*1_ versus the concentration *c*_*H*1*N*1_ for fixed concentrations of *c*_*RSV*–*A*2_ and (B) γ_*RSV*–*A*2_ versus the concentration *c*_*RSV*–*A*2_ for fixed concentrations of *c*_*H*1*N*1_ for the mixture H1N1 & RSV-A2 in saliva. All the dashed curves are the fitting based on the BET equation, and the black solid curves are the α–*c*_*A*_ relationship for the corresponding single virus in saliva. The plots of extracted *q* values based on the BET equation for (C) H1N1 (●) and H3N2 (▲) as a function of RSV concentration, and (D) RSV-A2 (blue) and RSV-B1 (red) as a function of the influenza concentration.

However, in saliva, the BET constants increase even more significantly. As shown in Figure 7C, H1N1 exhibits ${\mathrm{q̅}}_{H1N1}=2794$ , σ_*H*1*N*1_ = 3871 in the RSV-A2 mixture, and ${\mathrm{q̅}}_{H1N1}=2395$ , σ_*H*1*N*1_ = 2884 in the RSV-B1 mixture, representing 15–17.5 fold increases over the single-virus value of *q*_*H*1*N*1_ = 160. For H3N2, ${\mathrm{q̅}}_{H3N2}=205$ with σ_*H*3*N*2_ = 121 in the RSV-A2 mixture and ${\mathrm{q̅}}_{H3N2}=202$ with σ_*H*3*N*2_ = 153 in the RSV-B1 mixture, compared to the single-virus value of *q*_*H*3*N*2_ = 62.3, reflecting a 3.3-fold increase. Similar enhancements are observed for RSV viruses in saliva, as summarized in Figure 7D. RSV-A2 shows ${\mathrm{q̅}}_{RSV-A2}=164$ , σ_*RSV*–*A*2_ = 113 in the H1N1 mixture and ${\mathrm{q̅}}_{RSV-A2}=212$ , σ_*RSV*–*A*2_ = 363 in the H3N2 mixture, 7–9 times higher than the single-virus value of *q*_*RSV*–*A*2_ = 23. For RSV-B1, the enhancement is even more pronounced: ${\mathrm{q̅}}_{RSV-B1}=147$ , σ_*RSV*–*B*1_ = 90 in the H1N1 mixture and ${\mathrm{q̅}}_{RSV-B1}=863$ , σ_*RSV*–*B*1_ = 1547 in the H3N2 mixture, compared to a single-virus value of *q*_*RSV*–*B*1_ = 10. These correspond to increases of approximately 14.7- to 90-fold.

For 2VMs in saliva, the *k* parameter (Figure S5E,F) and corresponding *c* ^0^ values (Figure S5G,H) display greater variability compared to their water counterparts. Unlike in water, values no longer maintain consistent across concentrations for either virus component. Instead, we observe concentration-dependent fluctuations in *k*, with only a few cases showing relative stability: H3N2 throughout the H3N2 & RSV-B1 mixture series, H3N2 at concentrations ≥ 3,125 PFU/mL in H3N2 & RSV-A2 mixtures, and RSV-B1 at concentrations ≥ 781 PFU/mL in H1N1 & RSV-B1 mixtures. This increased variability likely reflects the complex interactions between virus particles and various biomolecules present in saliva. Despite these fluctuations, all calculated *c* ^0^ values consistently exceed 10^5^ PFU/mL, indicating that our highest experimental concentration remains below saturation for all virus types, even in the more complex saliva matrix.

These results demonstrate that the presence of a second virus and the composition of the surrounding medium, specifically water versus saliva, significantly impact virus–surface interactions, as reflected by changes in the BET constant q. In all four 2VMs, adsorption continued to follow BET-type behavior, yet the magnitude of q increased substantially compared to the corresponding single-virus systems. These enhancements are consistent across both aqueous and biological media and highlight the importance of considering coadsorption effects and matrix complexity in the quantitative modeling of virus detection.

The significant increases in BET constants observed in mixtures of influenza and RSV viruses can be attributed to several cooperative and competitive adsorption mechanisms, driven by the two virus families’ distinct physical and biochemical properties. Influenza viruses (e.g., H1N1 and H3N2) are relatively small (80–120 nm) and possess densely packed surface glycoproteins, hemagglutinin and neuraminidase, facilitating host-cell binding.^54^ In contrast, RSV strains (A2 and B1) are larger (120–300 nm) and feature long, flexible, and heavily glycosylated G and F proteins.^54^ These differences in size, surface composition, and flexibility enable intervirus and virus–surface interactions that extend beyond simple, independent physisorption.

One likely mechanism is competitive and cooperative binding.^55^ RSV’s extended glycoproteins may modify or precondition the surface, exposing additional binding domains that enhance the subsequent adsorption of influenza. Conversely, influenza’s compact and charged surface features could disrupt hydration layers or reorganize the local environment in ways that increase RSV binding. These microenvironmental changes may lead to the formation of new high-affinity sites or facilitate more efficient occupation of existing ones, increasing overall surface binding strength. Increased surface coverage and multilayer formation are other plausible contributors. The larger, deformable RSV virions can spread out and occupy more surface area, potentially allowing influenza virions to adsorb on top of or between RSV particles, creating interdigitated or stacked multilayers. This layering effect would contribute to the observed elevation in BET constants and further support the role of multilayer physisorption in mixed-virus systems. Electrostatic and steric interactions may also play a critical role.^56^ Variations in surface charge density and glycoprotein architecture between the two viruses could generate asymmetric charge distributions, resulting in favorable electrostatic interactions between coadsorbed virions. For example, RSV’s higher surface charge or glycoprotein flexibility might attract influenza virions or stabilize their binding through modifications in the electric double layer. Hydration shell restructuring and clustering effects further enhance these interactions.^57^ RSV’s heavily glycosylated surface maintains a thick hydration shell that may be partially disrupted by influenza, enabling more effective surface contact. Virus–virus clustering could also promote favorable orientations for adsorption, enhancing contact area and contributing to higher adsorption strength. Dynamic competition for surface sites or hot-spot locations can add another layer of complexity. While both viruses may compete for similar binding hot-spot regions, their interaction may be sequential or spatially partitioned. For instance, RSV could initially dominate surface regions, leaving high-affinity pockets for influenza, which would then bind more strongly. This dynamic partitioning can yield elevated*q* values that do not simply reflect the additive effects of two independent adsorbates. Importantly, the classical BET model assumes noninteracting adsorbates, an assumption that may fail in binary virus systems. Intervirus interactions, whether through nonspecific contacts or shared recognition elements, introduce nonlinear effects that manifest as disproportionately high BET constants in mixtures. These results demonstrate the limitations of traditional single-analyte adsorption models when applied to complex biological systems.

These mechanistic insights have important implications for quantitative virus detection. The apparent discrepancies between adsorption behavior in virus mixtures and single-virus suspensions challenge the validity of standard calibration curves derived from single-virus data. In single-virus systems, the BET constant and corresponding adsorption dynamics reflect only the interaction between the virus and the substrate. However, in mixtures, virus–virus interactions, competition for binding sites, and altered aggregation states significantly modulate these dynamics. Consequently, calibration curves based solely on single-virus adsorption are unlikely to represent the concentration–signal relationship in mixed-virus samples accurately. This limitation has practical consequences in surface-based biosensing platforms, such as SERS, electrochemical sensors, and immunoassays, that rely on surface adsorption for signal generation. Misapplication of single-virus calibration standards in the context of virus mixtures may lead to substantial overestimation or underestimation of virus concentrations, reducing diagnostic accuracy and reliability. To address this challenge, it is essential to develop mixture-aware quantification strategies.^30^ These could include multicomponent adsorption models, machine learning-based regression frameworks trained on mixed-virus data sets, or empirically derived standard curves from controlled coinfection experiments. By incorporating the effects of virus–virus and virus–surface interactions, such approaches will provide more robust and accurate quantification tools for real-world diagnostic applications.

### 3.6 Real-World Implementation

While our primary objective was to characterize virus-substrate binding mechanisms using the BET framework, the mathematical form of our model enables exploration of its potential for concentration prediction applications. To evaluate this secondary capability, we performed comprehensive validation analysis using an independent data set of 1,521 individual spectra across 10 virus types in saliva.^30^ Critically, these validation spectra represent genuine unknown samples: although they contain the same virus types in identical saliva backgrounds as our training data, their concentrations were completely excluded from the original BET model fitting process. This experimental design enables rigorous testing of predictive capability under realistic conditions where sample concentrations are genuinely unknown.

The validation methodology follows a straightforward process that mimics practical diagnostic scenarios. For each validation spectrum, we performed spectral decomposition using the same virus reference spectra and background components employed in the original model development, yielding the coefficient ratioα for the target spectrum. We then applied the fitted BET equation for the corresponding virus to predict concentration by solving for*c*_*A*_ given the measuredα value. Finally, we compare the predicted concentration*c*_*pre*_ to the actual concentration*c*_*act*_.

Figure 8 shows representative scatter plots of *c*_*pre*_ versus *c*_*act*_ in log–log scale from our comprehensive validation analysis across all 10 virus types. The complete validation data set reveals a striking inverse correlation *R* ^2^ and binding strength (*q* parameter), establishing clear performance boundaries for quantitative applications. To illustrate this relationship, we selected the four most extreme examples based on R^2^ values: the two highest-performing viruses—Flu B (*R* ^2^ = 0.937, *q* = 4) and HMPV-A (*R* ^2^ = 0.967, *q* = 11.35)—exhibit excellent quantitative utility with data points clustered tightly around the ideal prediction line (Figure 8A,B). In contrast, the two poorest performers—CoV-229E (*R* ^2^ = 0.025, *q* = 20.18) and H1N1 (*R* ^2^ = −0.193, *q* = 160)—show systematic prediction problems or complete quantitative failure (Figure 8C,D). Results for other viruses can be found in Figure S9, arranged in order of increasing *q* values.

**Figure 8:**
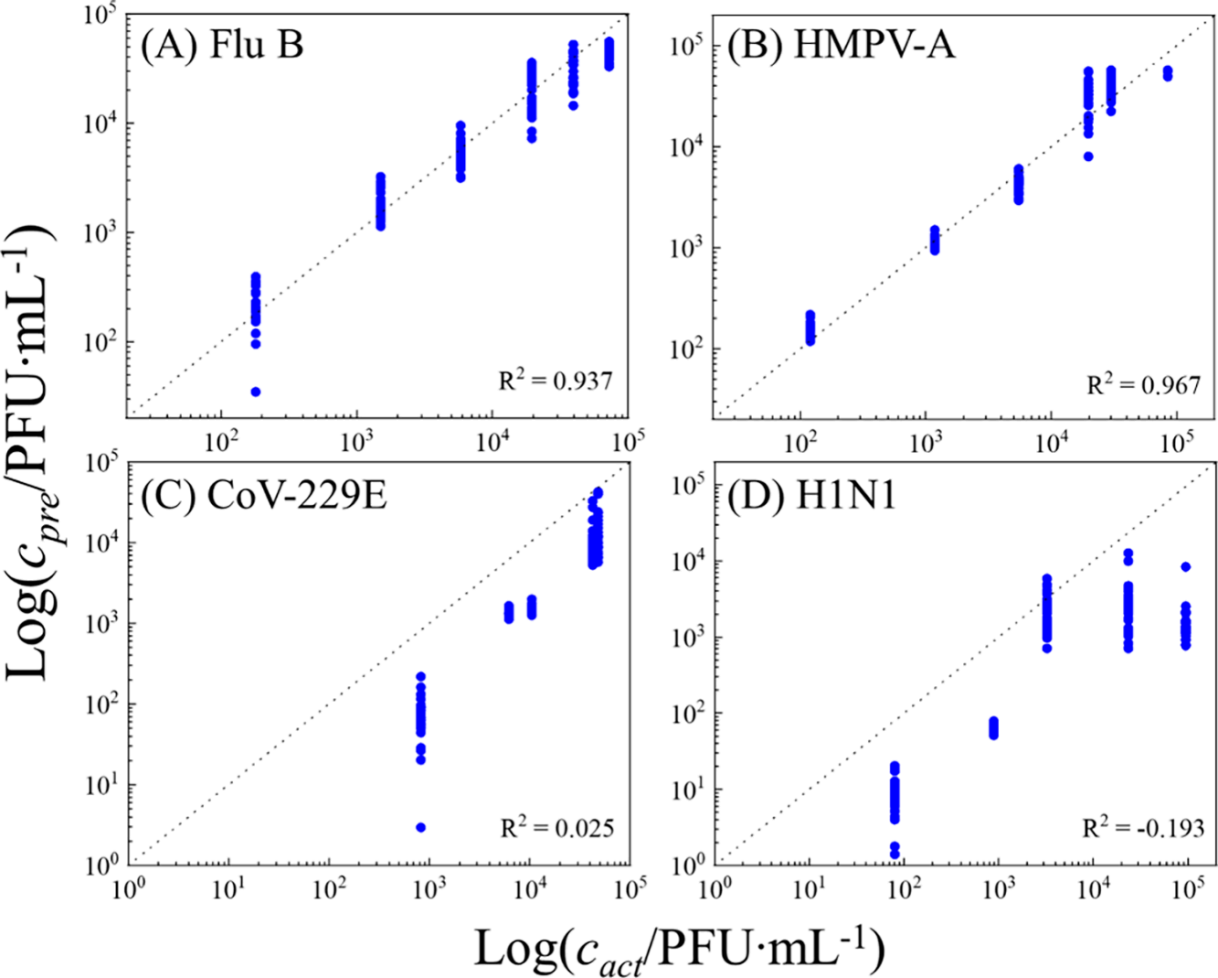
Scatter plots of predicted concentrations *c*_*pre*_ versus actual concentrations *c*_*act*_ in log-log scale using the BET model for each individual virus spectrum in saliva with unknown samples not included in the original model fitting. The diagonal dashed line represents perfect prediction. (A) Flu B, (B) HMPV-A, (C) CoV-229E, (D) H1N1.

The complete validation results demonstrate that viruses with*q* ≤ 11.35, including Flu B, Ad5, RSV-B1, HMPV-B, and HMPV-A, all achieve *R* ^2^ values above 0.82, enabling reliable concentration determination. Conversely, viruses with *q* ≥ 20.18, including CoV-229E, RSV-A2, H3N2, CoV-OC43, and H1N1, show progressively declining performance with systematic underprediction, concentration-dependent errors, or complete model failure. This establishes a clear performance threshold around*q* between 11.35 and 20.18, where the BET framework transitions from providing both mechanistic insights and quantitative utility to serving purely as a characterization tool.

The systematic limitations for high-q viruses arise from fundamental mathematical properties of the BET equation rather than experimental inadequacies. As *q* increases, the BET isotherm (eq 16) exhibits characteristic shape changes: while small*q* values show relatively linear relationships in the low-to-moderate concentration range, increasing *q* causes the curve to become increasingly flat in the low concentration region, with most response occurring only at very high concentrations approaching *c*_*A*_^0^ = 1/*k*. For strongly binding viruses, the curve becomes nearly insensitive to concentration changes across most experimental ranges, making quantitative applications ill-conditioned. During parameter fitting, the algorithm cannot adequately compensate for systematic deviations at low concentrations, resulting in compromised parameters that systematically underestimate concentrations during prediction. These physical limitations reflect violations of BET assumptions when binding strength becomes excessive, as surface heterogeneity dominates and strongly binding viruses preferentially occupy highest-energy sites, creating concentration-dependent binding behavior not captured by the uniform site model.

This boundary analysis establishes when our BET framework can serve dual purposes versus when it functions purely as a mechanistic tool. For moderate-binding viruses (*q* ≤ 11.35), the framework enables both fundamental characterization of binding behavior and accurate concentration determination under single-virus conditions. For strongly binding viruses, the framework successfully captures complex adsorption mechanisms and provides valuable insights into virus-surface interactions, but quantitative applications become unreliable due to mathematical constraints inherent to the BET formalism. Additionally, reliable quantitative prediction is inherently restricted to single-virus scenarios because binary-virus BET models require predefined concentrations for one component, whereas simultaneous determination of multiple unknown concentrations remains challenging.

Importantly, these quantitative boundaries do not limit the framework’s primary contribution in characterizing virus-surface interactions across all binding regimes. Our BET modeling successfully captures competitive and cooperative adsorption behaviors for all virus types and mixture conditions, providing fundamental insights essential for understanding biosensor performance, designing antiviral surfaces, and developing mixture-aware analytical strategies. The demonstrated quantitative utility for specific virus types represents an additional capability that emerges from the mechanistic framework under favorable mathematical conditions.

While our current study establishes a robust mathematical framework linking the SERS decomposition coefficientα to virus surface coverage via the BET model, we recognize the importance of cross-validation using independent experimental techniques. The current correlation relies on spectral decomposition and curve fitting with high fidelity (*R* ^2^ > 0.96 for most cases), which supports internal consistency. Moreover, boundary validation using unseen concentration samples showed strong quantitative agreement within identified performance regimes. Future work will aim to further validate virus adsorption quantities using conventional surface binding assays such as quartz crystal microbalance (QCM), ELISA surface quantification, or direct counting via fluorescence-tagged virions to strengthen the linkage between SERS-derived α values and actual virus surface coverage.

## 4 Conclusions

This study provides a detailed and quantitative understanding of virus adsorption and coadsorption behaviors on nanostructured surfaces using SERS and a modified BET adsorption model. By systematically analyzing 12 respiratory viruses, including influenza A/B, RSV A/B, coronaviruses, adenovirus, and human metapneumoviruses, in both water and saliva, we demonstrate that virus–surface interactions are governed predominantly by multilayer physisorption. All virus adsorption profiles conform to Type II isotherms, and the BET constant*q* serves as a sensitive parameter for quantifying the strength of surface binding. Our results reveal that adsorption strength varies substantially across virus types, reflecting particle size, surface glycoprotein architecture, and envelope composition differences. Virus mixtures exhibit significantly enhanced adsorption behavior compared to their single-virus counterparts. In many cases, BET constants in 2VMs increased by factors of 4 to 25, suggesting strong cooperative or competitive interactions that alter surface binding dynamics. The surrounding medium further modulates these effects: saliva introduces additional complexity through nonspecific biomolecular interactions, leading to virus-specific enhancements or suppressions in adsorption strength relative to water.

Mechanistically, these observations can be attributed to a combination of factors, including surface restructuring, multilayer formation, electrostatic interactions, hydration shell dynamics, and intervirus competition for binding sites. Significantly, these interactions violate the assumptions of classical adsorption models that treat adsorbates as independent entities, underscoring the need for more sophisticated models that account for virus–and matrix–virus interactions. From an analytical standpoint, our findings demonstrate that calibration curves based on single-virus systems are not reliably transferable to complex, multivirus environments. This highlights a critical limitation in conventional biosensing strategies and calls for developing mixture-aware calibration frameworks. Incorporating spectral decomposition, multicomponent BET fitting, and machine learning models trained on mixed-virus data sets will be essential for achieving accurate and robust virus quantification in real-world diagnostic and surveillance contexts.

These insights are highly relevant for practical applications. In clinical diagnostics, especially during respiratory outbreaks, coinfections are common and often undetected by conventional single-pathogen assays. Our validation analysis using unknown samples revealed that quantitative utility depends critically on binding strength: moderate-binding viruses (*q* ≤ 11.35) enable reliable concentration determination, while strongly binding viruses (*q* ≥ 20.18) exceed the mathematical boundaries of BET-based prediction. The BET constants extracted through our model could guide the design of next-generation SERS-based sensors, antiviral coatings, or sampling protocols optimized for specific virus types and physiological media. Moreover, this methodology offers a framework for evaluating and calibrating biosensors in the presence of complex biological interferences, such as those found in saliva or wastewater, making it relevant to point-of-care diagnostics, environmental surveillance, and public health response.

The methodological framework established here offers several impactful avenues for implementation. First, the combination of SERS and BET modeling can be integrated into developing multiplexed, surface-based diagnostic platforms for point-of-care applications, particularly in detecting coinfections where multiple respiratory viruses are present. Second, the mechanistic insights gained from BET constants can guide the design of next-generation antiviral surfaces and coatings by targeting physicochemical properties that minimize virus adsorption and retention. Third, the approach can be extended to study adsorption behavior in other biological matrices, such as serum, nasal fluid, or wastewater, making it highly relevant for environmental virology, public health monitoring, and epidemiological surveillance. Finally, the data set and modeling tools developed in this work can be used to train AI-driven diagnostic algorithms, with our validation showing reliable concentration estimation for single moderate-binding viruses, while mixture quantification remains a challenge requiring further development. This will enhance the accuracy and scalability of SERS-based biosensors in clinical, field, and resource-limited settings. Overall, this study deepens our fundamental understanding of virus–surface interactions and lays the groundwork for translating SERS and adsorption modeling into practical, high-impact biosensing technologies.

## Supplementary Material



## Data Availability

The data used in this manuscript are available athttps://github.com/jimcui3/Virus-adsorption-BET-fitting

## Notes

The Supporting Information is available along with the manuscript, including Information about single viruses and binary virus mixtures; Linear decomposition results of 2VMs in water, SVs in saliva, and 2VMs in saliva; BET fitting parameters and results for SVs in water and saliva; BET fitting results and parameters for 2VMs in water and saliva (PDF)
