# Supplementary material for: Quantitative Analysis of Virus Adsorption and Co-adsorption Behavior Using BET Modeling and SERS Spectroscopy
Source: J Phys Chem A. 2025 Aug 25;129(35):8204–19. doi: 10.1021/acs.jpca.5c03375 (PMC12415823; doi:10.1021/acs.jpca.5c03375)
Supplement: Supplementary file 1 [file jp5c03375_si_001.pdf]

Supporting Information

Quantitative Analysis of Virus Adsorption and Co-adsorption Behavior Using BET Modeling and SERS Spectroscopy

Jiaheng Cui [a], Yanjun Yang [b] *, Amit Kumar [b], Jackelyn Murray [c], Les Jones [c], Xianyan Chen [d], Ralph A. Tripp [c], and Yiping Zhao [b]*

[a] School of Electrical and Computer Engineering, College of Engineering, The University of Georgia, Athens, GA, USA 30602

[b] Department of Physics and Astronomy, The University of Georgia, Athens, GA, USA 30602

[c] Department of Infectious Diseases, College of Veterinary Medicine, The University of Georgia, Athens, GA, USA 30605

[d] Department of Epidemiology & Biostatistics, College of Public Health, The University of Georgia, Athens, GA, USA 30602

* Corresponding Author: E-mail: yanjunyang@uga.edu, zhaoy@uga.edu

Section S1. Information about single viruses and binary virus mixtures.

Table S1. Information about single viruses and binary virus mixtures in water and saliva.

Category	Virus species in water	Virus species in saliva
**Single viruses (SVs)**	Ad5	Ad5
	CoV-2	-
	CoV-229E	CoV-229E
	CoV-2 B1	-
	CoV-OC43	CoV-OC43
	Flu B	Flu B
	H1N1	H1N1
	H3N2	H3N2
	HMPV-A	HMPV-A
	HMPV-B	HMPV-B
	RSV-A2	RSV-A2
	RSV-B1	RSV-B1
**Binary virus mixtures (2VMs)**	H1N1 & RSV-A2	H1N1 & RSV-A2
	H1N1 & RSV-B1	H1N1 & RSV-B1
	H3N2 & RSV-A2	H3N2 & RSV-A2
	H3N2 & RSV-B1	H3N2 & RSV-B1

Section S2. Material characterization of the AgNR substrates.

To evaluate the reproducibility and enhancement uniformity of the AgNR SERS substrates, 5 μL of 10^{-5} M BPE was drop-cast onto a representative substrate, and SERS spectra were collected from 20 randomly selected positions. As shown in **Figure S1**, the spectral profiles were highly consistent, and the calculated relative standard deviations (RSDs) for major Raman peaks at 1200 cm^{-1}, 1610 cm^{-1}, and 1640 cm^{-1} were 4.6%, 4.5%, and 4.0%, respectively, confirming excellent signal uniformity and substrate quality.

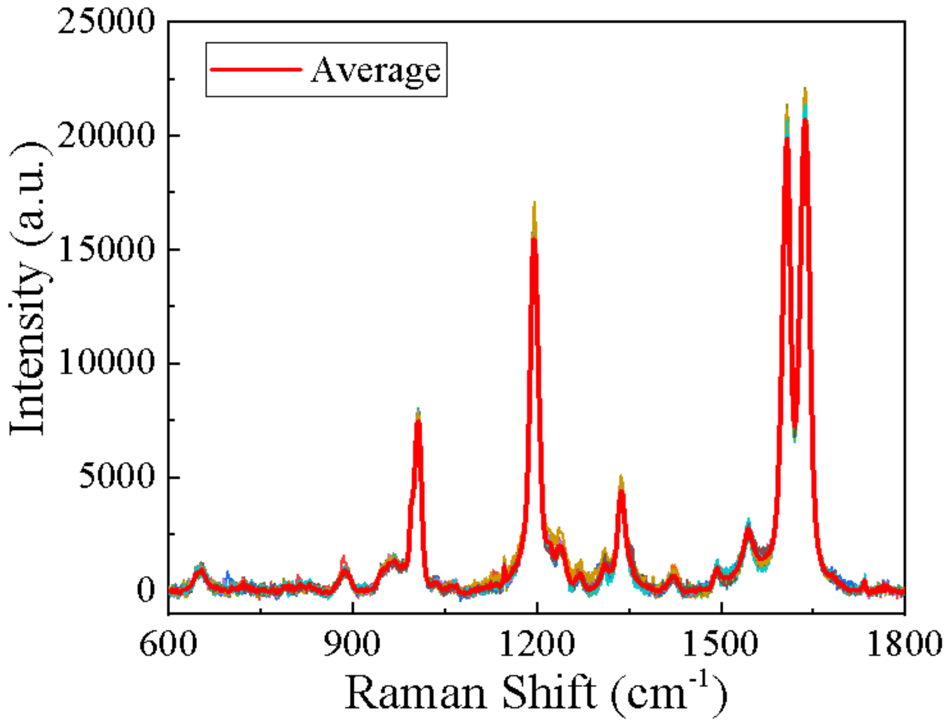

Figure S1. SERS spectra of 10^{-5} M BPE collected from 20 randomly selected positions on the AgNR substrate, with the average spectrum shown in red.

To justify the use of 785 nm excitation wavelength, we conducted reflectance measurements on the same substrate. As shown in **Figure S2**, a broad reflectance dip was observed near 785 nm, indicative of localized surface plasmon resonance coupling. This spectral feature confirms strong near-field enhancement at the chosen excitation wavelength, aligning with prior reports on GLAD-fabricated AgNRs.

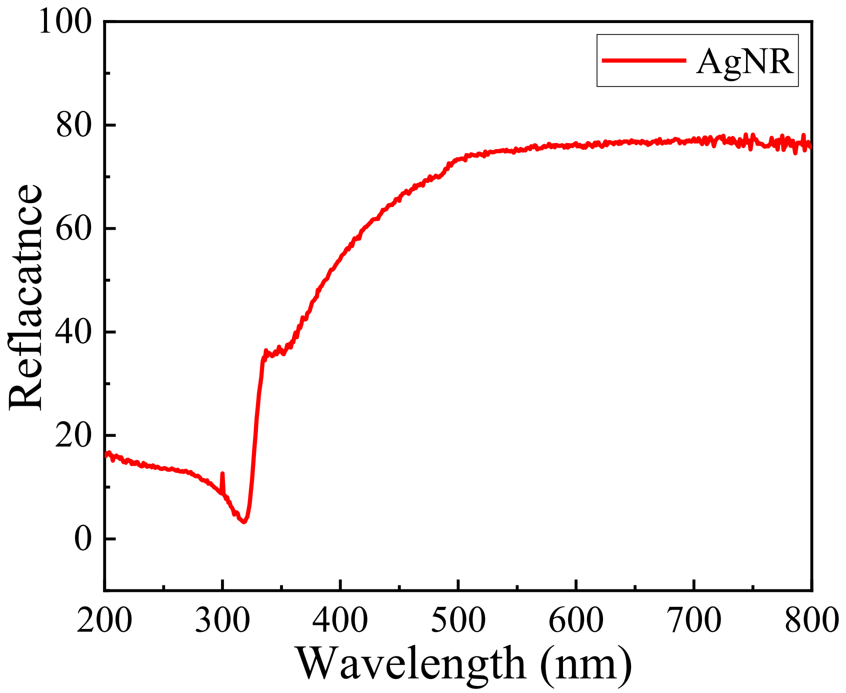

Figure S2. Reflectance spectrum of the AgNR substrate showing the plasmonic resonance dip near 785 nm excitation wavelength.

Section S3. Linear decomposition results of 2VMs in water, SVs in saliva, and 2VMs in saliva. Average and reconstructed spectra for every concentration of all SVs and each concentration combination of 2VMs, together with corresponding fitting visualizations for SVs and 2VMs in both water and saliva, can be accessed at the following GitHub repository: https://github.com/jimcui3/Virus-adsorption-BET-fitting

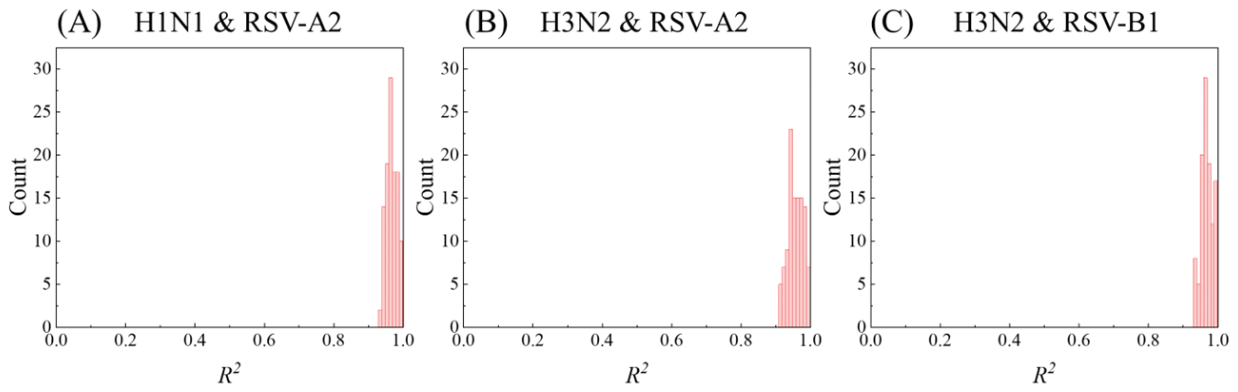

Figure S3. Histogram illustrating the distribution of R^2 values for the (A) H1N1 & RSV-A2, (B) H3N2 & RSV-A2, (C) H3N2 & RSV-B1 2VMs in water.

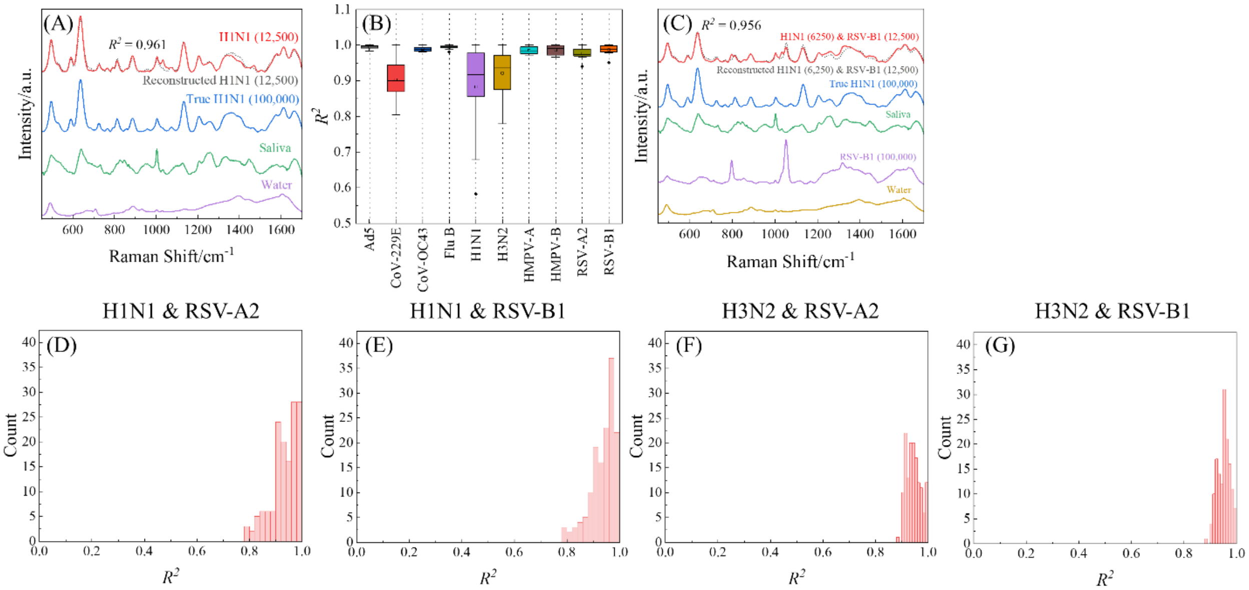

Figure S4. Representation of linear decomposition fitting and goodness-of-fit evaluation for SVs spectra and 2VMs in saliva. (A) True spectrum (red) and reconstructed spectrum (grey dashed curve) of H1N1 virus at 12,500 PFU/mL, alongside reference spectra for H1N1 (10^5 PFU/mL, blue), saliva (green), and water (purple). (B) Boxplot summarizing R^2 values for all single-virus spectra, with whiskers representing ± 1.5 interquartile ranges (IQR), box boundaries indicating the 25th and 75th percentiles, center lines showing the median, and dots marking mean values. (C) True spectrum (red) and reconstructed spectrum (grey dashed curve) of the 2VM H1N1 (6,250 PFU/mL) and RSV-B1 (12,500 PFU/mL), with reference spectra for H1N1 (10^5 PFU/mL, blue), RSV-B1 (10^5 PFU/mL, purple), saliva (green), and water (brown). Histogram illustrating the distribution of R^2 values for the (D) H1N1 & RSV-A2, (E) H1N1 & RSV-B1, (F) H3N2 & RSV-A2, and (G) H3N2 & RSV-B1 2VM.

Section S4. BET fitting parameters and results for SVs in water and saliva.

Table S2. A summary table of the R^2 and parameters from BET models fitted to single-virus data acquired in both water and saliva backgrounds, c^0 (estimated saturated concentration) is calculated using $1/k$. The viruses are ordered by ascending q.

Water						Saliva					
**Virus Name**	R^2	q	A	k	c^0 (PFU/mL)	**Virus Name**	R^2	q	A	k	c^0 (PFU/mL)
H3N2	0.998	11.854	3.698	1.63×10^{-5}	61,350	Flu B	0.993	4	10.5	1.7×10^{-5}	58,824
CoV-2 B1	0.985	14.53	4.95	1.36×10^{-5}	73,529	Ad5	0.999	5.5	21	2.3×10^{-5}	43,478
H1N1	0.987	19.925	2.342	8.10×10^{-6}	123,457	RSV-B1	0.995	10	8.41	1.5×10^{-5}	66,667
HMPV-B	0.994	20.528	6.887	1.19×10^{-5}	84,034	HMPV-B	0.995	10.31	20.72	1.4×10^{-5}	71,429
HMPV-A	0.995	21.142	4.669	1.37×10^{-5}	72,993	HMPV-A	0.982	11.35	8.41	1.6×10^{-5}	62,500
CoV-OC43	0.998	21.98	3.92	1.38×10^{-5}	72,464	CoV-229E	0.933	20.18	1.5	1.77×10^{-5}	56,497
Ad5	0.998	24.1	4.37	1.64×10^{-5}	60,976	RSV-A2	0.987	23.15	2.48	1.64×10^{-5}	60,976
CoV-229E	0.943	36.175	9.998	1.50×10^{-5}	66,667	H3N2	0.762	62.31	3	5.1×10^{-6}	196,078
RSV-B1	0.985	39.752	3.451	8.48×10^{-6}	117,925	CoV-OC43	0.925	85	1.9	1.82×10^{-5}	54,945
Flu B	0.988	98.609	3.914	1.45×10^{-5}	68,966	H1N1	0.800	160	12.5	1.55×10^{-5}	64,516
CoV-2	0.972	104.473	9.892	1.58×10^{-5}	63,291	CoV-2 B1	-	-	-	-	-
RSV-A2	0.964	120.418	2.852	5.70×10^{-6}	175,439	CoV-2	-	-	-	-	-

Section S5. BET fitting results and parameters for 2VMs in water and saliva.

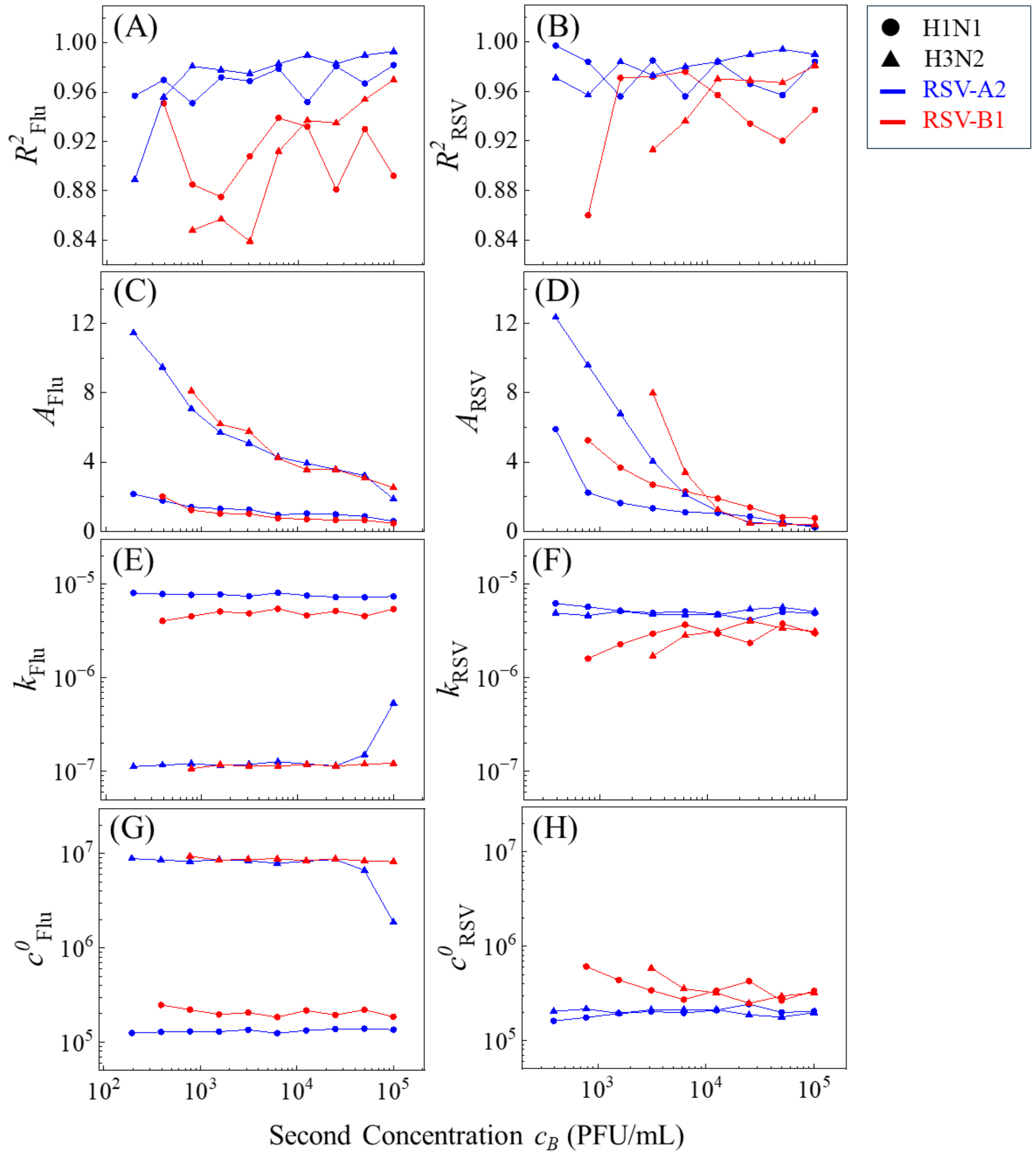

Figure S5. Analysis of virus binding parameters of 2VMs in water using the BET equation. Left column (A, C, E, G): Parameters for influenza viruses H1N1 (●) and H3N2 (▲) plotted against RSV concentration. Right column (B, D, F, H): Parameters for RSV-A2 (blue) and RSV-B1 (red) plotted against influenza concentration. Row-wise parameters: (A, B) R^2 values, (C, D) A, (E, F) k, and (G, H) c^0.

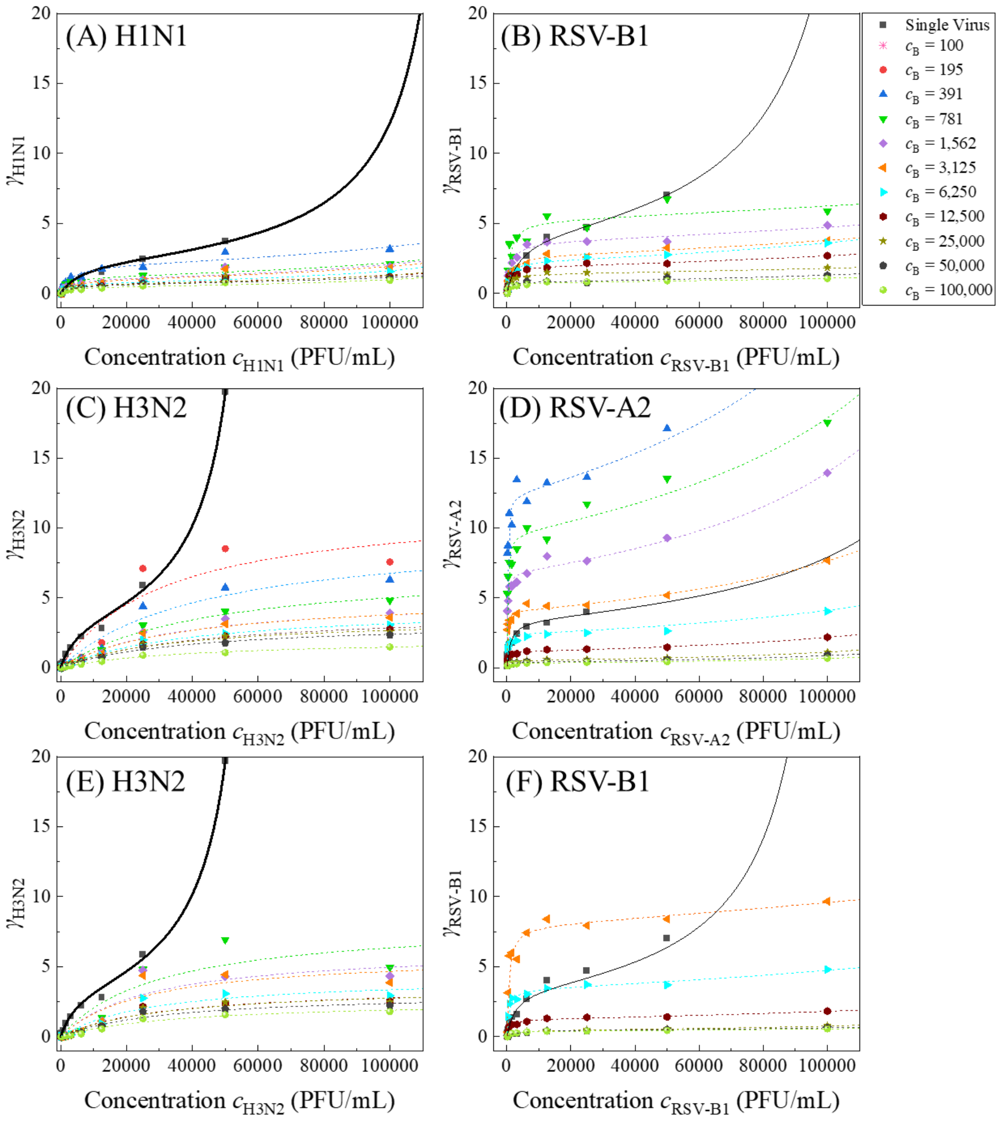

Figure S6. The plots of (A) γ_{H1N1} versus the concentration c_{H1N1} for fixed concentrations of c_{RSV-B1} and (B) γ_{RSV-B1} versus the concentration c_{RSV-B1} for fixed concentrations of c_{H1N1} for the mixture H1N1 & RSV-B1 in water, (C) γ_{H3N2} versus the concentration c_{H3N2} for fixed concentrations of c_{RSV-A2} and (D) γ_{RSV-A2} versus the concentration c_{RSV-A2} for fixed concentrations of c_{H3N2} for the mixture H3N2 & RSV-A2 in water, (E) γ_{H3N2} versus the concentration c_{H3N2} for fixed concentrations of c_{RSV-B1} and (F) γ_{RSV-B1} versus the concentration c_{RSV-B1} for fixed concentrations of c_{H3N2} for the mixture H3N2 & RSV-B1 in water. All the dashed curves are the fitting based on the BET equation, and the black solid curves are the $\alpha - c_A$ relationship for the corresponding single virus in water.

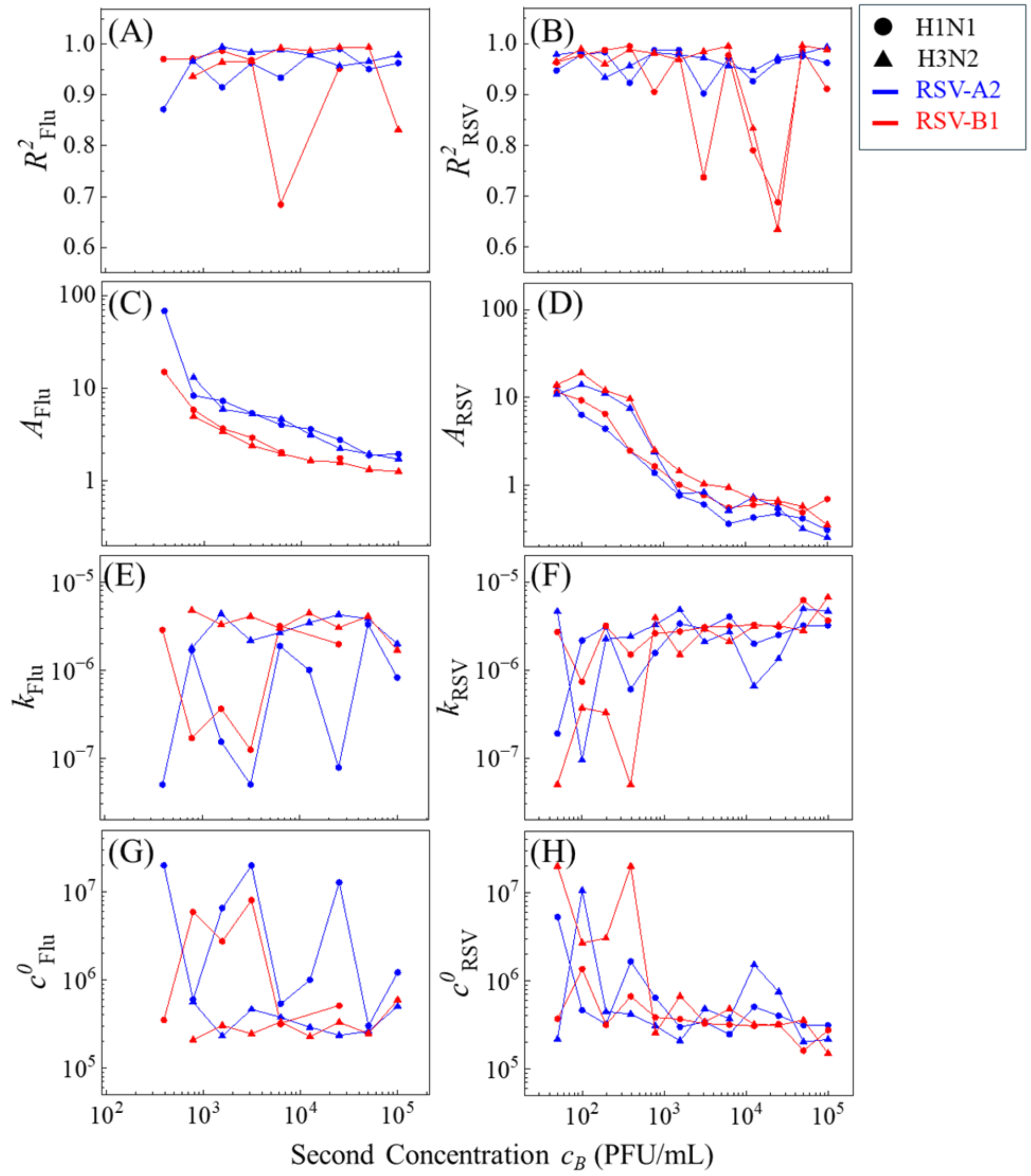

Figure S7. Analysis of virus binding parameters of 2VMs in saliva using the BET equation. Left column (A, C, E, G): Parameters for influenza viruses H1N1 (●) and H3N2 (▲) plotted against RSV concentration. Right column (B, D, F, H): Parameters for RSV-A2 (blue) and RSV-B1 (red) plotted against influenza concentration. Row-wise parameters: (A, B) R^2 values, (C, D) A, (E, F) k, and (G, H) c^0.

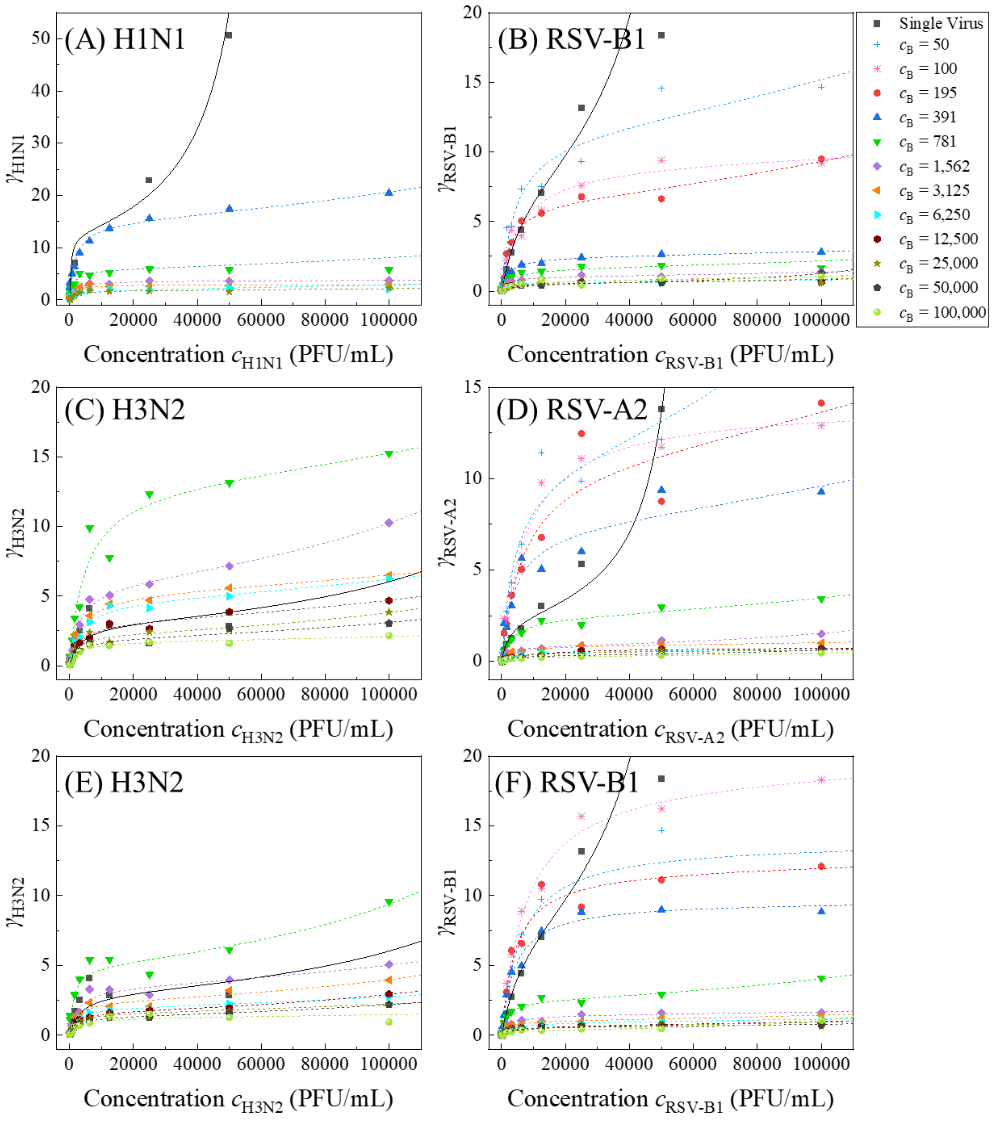

Figure S8. The plots of (A) γ_{H1N1} versus the concentration c_{H1N1} for fixed concentrations of c_{RSV-B1} and (B) γ_{RSV-B1} versus the concentration c_{RSV-B1} for fixed concentrations of c_{H1N1} for the mixture H1N1 & RSV-B1 in saliva, (C) γ_{H3N2} versus the concentration c_{H3N2} for fixed concentrations of c_{RSV-A2} and (D) γ_{RSV-A2} versus the concentration c_{RSV-A2} for fixed concentrations of c_{H3N2} for the mixture H3N2 & RSV-A2 in saliva, (E) γ_{H3N2} versus the concentration c_{H3N2} for fixed concentrations of c_{RSV-B1} and (F) γ_{RSV-B1} versus the concentration c_{RSV-B1} for fixed concentrations of c_{H3N2} for the mixture H3N2 & RSV-B1 in saliva. All the dashed curves are the fitting based on the BET equation, and the black solid curves are the $\alpha - c_A$ relationship for the corresponding single virus in water.

Section S6. BET fitting as a prediction model of unknown samples in saliva.

As described in **Section 3.6**, we performed comprehensive validation analysis using 1,521 individual spectra across 10 virus types, which were excluded from the original BET model fitting process. [1] **Figure S9** illustrates predicted concentrations c_{pre} versus actual concentrations c_{act} for the six virus types not featured in **Figure 8**, arranged in order of increasing q values: Ad5 (**Figure S9A**), RSV-B1 (**Figure S9B**), HMPV-B (**Figure S9C**), RSV-A2 (**Figure S9D**), H3N2 (**Figure S9E**), and CoV-OC43 (**Figure S9F**).

The underprediction phenomenon visible in **Figure S9D-F** arises from a fundamental mismatch between the fitted BET curve and experimental data distribution. In typical experiments, data points tend to fall below the theoretical BET curve at low concentrations due to surface heterogeneity effects. During fitting, the algorithm attempts to minimize residuals by adjusting parameters. For low-q viruses, this adjustment process can adequately compensate for the systematic deviation, as evidenced by the tight clustering around the ideal line in **Figure S9A-C**. However, for high-q viruses, the BET curve's reduced sensitivity means that even large parameter adjustments cannot bring the fitted curve close to the low-concentration data points. The fitting process becomes dominated by minimizing residuals at higher concentrations, where the BET curve finally shows significant response. This mathematical constraint forces the fitted parameters to represent a compromise that inadequately describes the low-concentration behavior. When this compromised model is used for prediction, it systematically underestimates concentrations because it cannot capture the true binding strength at low concentrations where the strongest binding sites dominate the response.

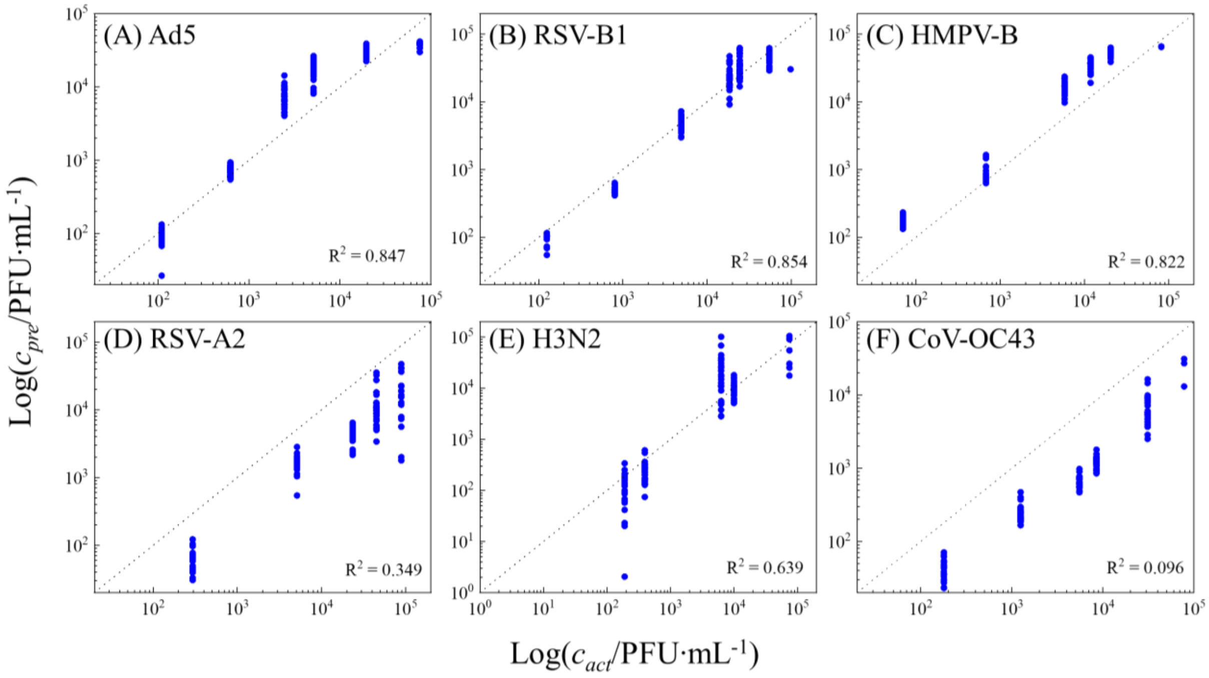

Figure S9. Scatter plots of predicted concentrations c_{pre} versus actual concentrations c_{act} using the BET model for each individual virus spectrum in saliva with unknown samples excluded in the original model fitting, arranged in order of increasing q values. The diagonal dashed line represents perfect prediction. (A) Ad5, (B) RSV-B1, (C) HMPV-B, (D) RSV-A2, (E) H3N2, (F) CoV-OC43.

The failure of BET modeling for high-q viruses reflects several physical limitations that become prominent when binding strength exceeds the model's assumptions. Surface heterogeneity becomes dominant when $q \geq 20.18$, as strongly binding viruses preferentially occupy the highest-energy sites, creating concentration-dependent binding behavior that violates BET assumptions of uniform sites. The extremely high q values (62-160) suggest that some viruses interact with specific, highly favorable surface sites that are not representative of the average surface energy. Additionally, kinetic limitations may affect viruses with $q > 50$, where binding becomes so strong that desorption becomes negligible, violating the BET assumption of rapid equilibrium. This is particularly relevant for H3N2 ($q = 62.31$), which shows unstable predictions with high variability, and CoV-OC43 ($q = 85$), and H1N1 ($q = 160$), which show near-complete model failure visible. The BET model also assumes that multilayer formation can occur, but for very strongly binding viruses, the first layer may be so tightly bound that subsequent layers cannot form under typical experimental conditions, further invalidating the model assumptions.

These results establish clear guidelines for when our BET model can be used predictively when it serves purely as a binding characterization tool. The visual evidence in **Figure 8** and **Figure S9** clearly demonstrates that viruses with $q \leq 11.35$ show excellent predictive performance, enabling accurate concentration determination across typical experimental ranges. However, viruses with $q \geq 20.18$ show systematic problems ranging from bias to complete model failure, making them unsuitable for quantitative prediction. This finding establishes clear boundaries for potential diagnostic applications by defining when our model can be used predictively, while emphasizing that its primary value lies in binding characterization rather than quantitative prediction for the full range of virus types studied.

Reference:

(1) Yang, Y.; Cui, J.; Kumar, A.; Luo, D.; Murray, J.; Jones, L.; Chen, X.; Hülck, S.; Tripp, R. A.; Zhao, Y. Multiplex Detection and Quantification of Virus Co-Infections Using Label-free Surface-Enhanced Raman Spectroscopy and Deep Learning Algorithms. *ACS Sensors* **2025**, *10* (2), 1298-1311. DOI: 10.1021/acssensors.4c03209.
